# Hemocytes are essential for *Drosophila melanogaster* post-embryonic development, independent of control of the microbiota

**DOI:** 10.1242/dev.200286

**Published:** 2022-09-29

**Authors:** Holly N. Stephenson, Robert Streeck, Florian Grüblinger, Christian Goosmann, Alf Herzig

**Affiliations:** ^1^ Department of Cellular Microbiology, Max Planck Institute for Infection Biology, Charitéplatz 1, Berlin 10117, Germany; ^2^ Peninsula Medical School, Faculty of Health, University of Plymouth, Plymouth, Devon PL4 8AA, UK

**Keywords:** *Drosophila*, Hemocytes, Metamorphosis, Pupal lethal

## Abstract

Proven roles for hemocytes (blood cells) have expanded beyond the control of infections in *Drosophila*. Despite this, the crucial role of hemocytes in post-embryonic development has long thought to be limited to control of microorganisms during metamorphosis. This has previously been shown by rescue of adult development in hemocyte-ablation models under germ-free conditions. Here, we show that hemocytes have an essential role in post-embryonic development beyond their ability to control the microbiota. Using a newly generated strong hemocyte-specific driver line for the GAL4/UAS system, we show that specific ablation of hemocytes is early pupal lethal, even under axenic conditions. Genetic rescue experiments prove that this is a hemocyte-specific phenomenon. RNA-seq data suggests that dysregulation of the midgut is a prominent consequence of hemocyte ablation in larval stages, resulting in reduced gut lengths. Dissection suggests that multiple processes may be affected during metamorphosis. We believe this previously unreported role for hemocytes during metamorphosis is a major finding for the field.

## Teaser

**Highlighted Article:** Hemocyte ablation in *Drosophila melanogaster* with a novel strong hemocyte-specific driver causes pupal lethality under germ-free conditions.

## INTRODUCTION

*Drosophila melanogaster* is an important model to study both the immune and non-immune related functions of blood cells (hemocytes) (Mase et al., 2021). Plasmatocytes are macrophage-like cells (∼95% of larval hemocytes) that secrete signaling peptides, anti-microbial peptides, and extracellular matrix (ECM) proteins in addition to phagocytosing microorganisms and apoptotic cells (Olofsson and Page, 2005; Braun et al., 1998). Crystal cells (∼5% of larval hemocytes) express high levels of pro-phenoloxidases, which catalyze the extracellular production of melanin and toxic by-products upon cell lysis; essential for wound closure and immunity (Binggeli et al., 2014). Lamellocytes, rarely found in healthy larvae, transdifferentiate in large numbers from plasmatocytes to encapsulate large pathogens (Sinenko et al., 2011). Recent single-cell RNA-seq studies have shown greater heterogeneity in these cell types (Cattenoz et al., 2020; Tattikota et al., 2020; Coates et al., 2021).

Two waves of hematopoiesis occur in *Drosophila* development. Embryonic hemocytes originate from the head mesoderm; they are long-lived, many surviving into the adult stage (Tepass et al., 1994). Larval hematopoiesis occurs in the lymph gland and in hematopoietic pockets (HP), patches of sessile hemocytes associated with the larval cuticle. HP are the main source of increasing numbers of circulating hemocytes during larval development (Leitao and Sucena, 2015); whereas hemocytes from the lymph gland are released into circulation at early metamorphosis (Jung et al., 2005).

Genetic ablation studies that aimed to identify the importance of hemocytes in *Drosophila* were first performed over a decade ago (Charroux and Royet, 2009; Nehme et al., 2011; Defaye et al., 2009; Shia et al., 2009; Arefin et al., 2015). A 60-75% reduction in larval hemocyte numbers was achieved by hemocyte-specific expression of pro-apoptotic transgenes, ablating cells through programmed cell death. Multiple studies showed a reduction in eclosion of adult flies of up to 60%; interestingly however, eclosion rates were rescued when larvae were reared with antibiotics or under germ-free (GF) conditions (Arefin et al., 2015; Charroux and Royet, 2009; Defaye et al., 2009; Shia et al., 2009). This suggested control of microorganisms by hemocytes is crucial during metamorphosis, and that hemocyte functions beyond immunity are non-essential for post-embryonic development (Charroux and Royet, 2009; Shia et al., 2009; Arefin et al., 2015; Defaye et al., 2009). In contrast, ablation of embryonic hemocytes is embryonic lethal, independent of control of microorganisms (Defaye et al., 2009; Shia et al., 2009).

In this study, we designed an improved *Drosophila* hemocyte-specific larval and adult driver line, *Hml*^P2A^-GAL4. Using *Hml*^P2A^-GAL4-driven apoptosis, we almost completely ablated hemocytes in the larvae. We show for the first time that hemocytes are essential for the development of adult stage flies, independent of control of the microbiota. RNA-seq data show a striking upregulation of genes in the midgut of ‘hemoless’ larvae, and points to a crucial role of hemocytes in regulating intestinal development and beyond.

## RESULTS AND DISCUSSION

### *Hml*^P2A^-GAL4 is a hemocyte-specific driver

Currently, the most widely used hemocyte-specific driver in larvae and adults is *Hml*^Δ^-GAL4, which uses 840 bp of the *Hml* enhancer (Sinenko and Mathey-Prevot, 2004). To generate an optimized enhancer element, we included the first nine exons of *Hml* followed by a P2A self-cleaving sequence upstream of GAL4 (*Hml*^P2A^-GAL4) (Fig. S1). Transgenic flies were generated at two landing-sites, attP40 and attP2.

**Fig. 1.**
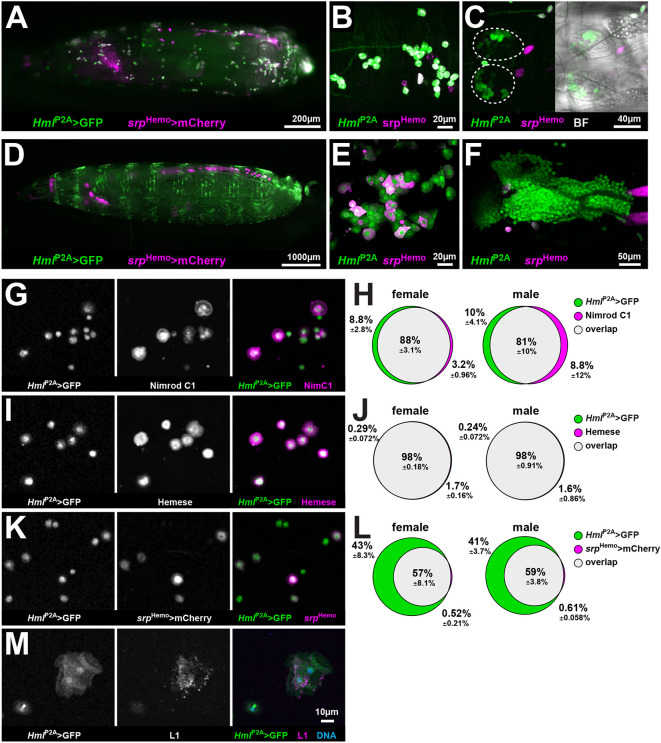
***Hml*^P2A^-GAL4 is a hemocyte-specific driver.** (A-F) Fluorescent microscopy of *Hml*^P2A^>GFP (attP40); *srp*^Hemo^>mCherry first instar (A-C) and WS-larvae (D-F). Whole animals (A,D); maximum projections of image stacks including peripheral hematopoietic pockets (B,E) and developing lymph gland (C,F). BF indicates brightfield images. (G-M) Fluorescent microscopy analysis (G,I,K,M) of hemocytes extracted from *Hml*^P2A^>GFP (attP40); *srp*^Hemo^>mCherry WS-larvae stained with plasmatocyte-specific (Nimrod C1), pan-hemocyte (Hemese) or lamellocyte-specific (L1) antibodies. Euler diagrams (H,J,L) show overlap of stainings and *Hml*^P2A^>GFP expression from *n*≥3 independent experiments, >30,000 cells analyzed. Overlap between L1 staining and *Hml*^P2A^>GFP expression was 95% (s.d. 6.8%) from a total of 211 cells analyzed from five independent experiments.

To analyze expression, we combined *Hml*^P2A^-GAL4 driving expression of EGFP (*Hml*^P2A^>GFP) with *srp*^Hemo^*-*QF2 driving mCherry (*srp*^Hemo^>mCherry) (Fig. 1; Fig. S2). *srp*^Hemo^*-*QF2 derives from the *serpent* enhancer and drives expression in embryonic and larval hemocytes (Gyoergy et al., 2018) as well as pericardial nephrocytes and Garland cells (Das et al., 2008). Consistent with the reported onset of *Hml* expression during first larval instar (Defaye et al., 2009), *srp*^Hemo^-positive (*srp*^Hemo^+) hemocytes in stage 17 embryos were mostly *Hml*^P2A^-negative (*Hml*^P2A^−), although faint expression was detected in some cells (Fig. S2B). In late first larval instar (Fig. 1A-C) *Hml*^P2A^ expression overlapped with *srp*^Hemo^ expression in peripheral HP with some hemocytes still *Hml*^P2A^− (Fig. 1B), and independent of *srp*^Hemo^ in the developing lymph gland (Fig. 1C). In late third larval instar wandering stage (WS) (Fig. 1D-F), *Hml*^P2A^+ hemocytes were a mixture of *srp*^Hemo^− and *srp*^Hemo^+ (Fig. 1E); lymph gland *Hml*^P2A^+ hemocytes were all *srp*^Hemo^− (Fig. 1F). This correlates with previous reports that *srp*^Hemo^ is expressed in hemocytes solely deriving from embryonic hematopoiesis (Bruckner et al., 2004). Overlap between *Hml*^P2A^ and *srp*^Hemo^ was restricted to hemocytes during the entire development and we did not observe *Hml*^P2A^ expression outside hemocytes (Fig. S2).

We next used hemocyte-specific antibodies to determine the coverage of *Hml*^P2A^ expression in hemocytes (Fig. 1G-L). The overlap between *Hml*^P2A^ expression and the plasmatocyte marker Nimrod C1 (Kurucz et al., 2007a) was >80% (Fig. 1G,H). Overlap with the pan-hemocyte marker Hemese (Kurucz et al., 2003) was ∼98% (Fig. 1I,J). Similar to the *in vivo* data, all *srp*^Hemo^+ hemocytes were *Hml*^P2A^+ in WS-larvae, whereas only ∼60% of *Hml*^P2A^+ hemocytes were *srp*^Hemo^+ (Fig. 1K,L). Very few lamellocytes were detected using the L1 antibody (Kurucz et al., 2007b) but, of those detected, 95% were *Hml*^P2A^+ (Fig. 1M).

### *Hml*^P2A^-GAL4 is a stronger driver than *Hml*^Δ^-GAL4

To compare the expression strength of *Hml*^P2A^-GAL4 and *Hml*^Δ^-GAL4 we assayed extracted hemocytes from *Hml*^P2A^>GFP and *Hml*^Δ^>GFP WS-larvae by flow cytometry (Fig. S3A). *Hml*^P2A^>GFP (attP2 and attP40) hemocytes showed ∼4-fold higher EGFP expression than *Hml*^Δ^>GFP hemocytes, which was consistent with microscopy of WS-larvae and adults (Fig. S3B,C).

**Fig. 2.**
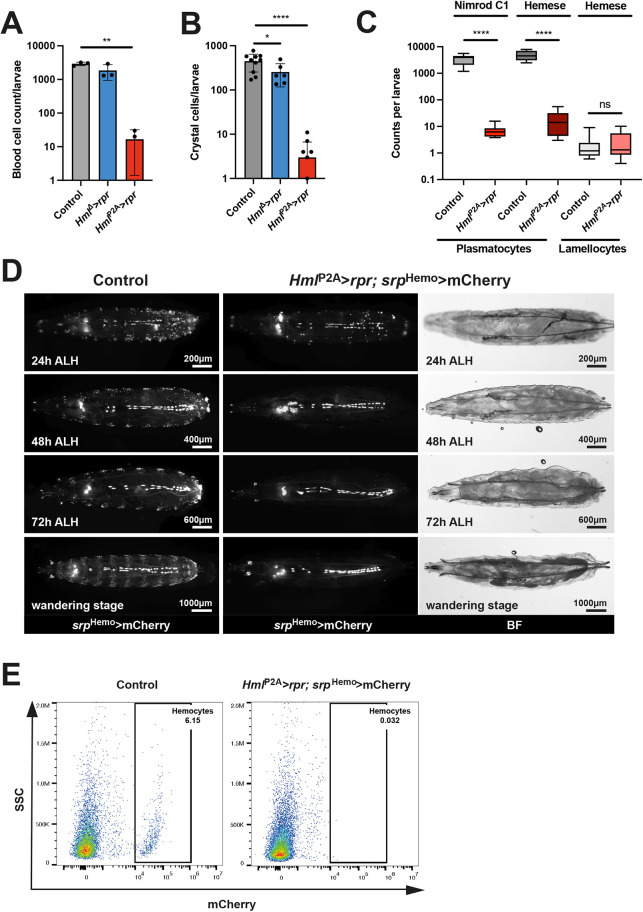
**Inducing apoptosis with *Hml*^P2A^-GAL4 ablates virtually all plasmatocytes and crystal cells.** (A-C) Hemocyte ablation in *Hml*^P2A^>*rpr* (attP2) WS-larvae. Whole blood cell counts by hemocytometer (A), crystal cell counts by whole mount microscopy (B) and quantification based on staining with plasmatocyte-specific (Nimrod C1) or pan-hemocyte (Hemese) antibodies (C). Hemese-positive lamellocytes were identified by cell morphology. Each dot represents average counts from five animals (A,C) or a single animal (B). One-way ANOVA (A,B) and two-tailed unpaired *t*-tests (C) were performed. Data are mean±s.d. (A,B). Box plots (C) show median values (middle bars) and first to third interquartile ranges (boxes); whiskers indicate minimum and maximum values. (D,E) Hemocyte ablation in *Hml*^P2A^>*rpr* (attP40); *srp*^Hemo^>mCherry larvae. (D) Whole-mount fluorescence microscopy of late first (24 h ALH), late second (48 h ALH), mid third (72 h ALH) and WS-larvae. BF indicates brightfield images. (E) Flow cytometry of cells from dissociated WS-larvae. Representative flow cytometry data from three independent experiments showing side scatter (SSC) and mCherry signals. Controls: attP2>*rpr* (A,B), yw>*rpr* (C), *Hml*^P2A^>GFP (attP40); *srp*^Hemo^>mCherry (D,E). *P<0.03; **P<0.002; ****P<0.0001. ns, not significant.

To compare the efficiency of hemocyte ablation between *Hml*^P2A^-GAL4 and *Hml*^Δ^-GAL4, we used them to drive expression of the pro-apoptotic gene *reaper* (*rpr*) or the mouse BCL2-associated X protein gene (*Bax*). Consistent with previous studies (Arefin et al., 2015; Charroux and Royet, 2009; Defaye et al., 2009; Shia et al., 2009), *Hml*^Δ^-GAL4 induced significant but incomplete ablation of total blood cells or crystal cells (Fig. 2A,B; Fig. S3D-F). *Hml*^P2A^-GAL4 (attP2) improved the efficiency of *rpr*-mediated ablation to >99% in both cases (Fig. 2A,B). We verified ablation of plasmatocytes by staining for Nimrod C1 and Hemese (Fig. 2C). We also identified Hemese-positive lamellocytes by cell morphology, but observed no depletion despite *Hml*^P2A^ expression in lamellocytes (Fig. 1M). In contrast to previous studies with *Hml*^Δ^-GAL4, we neither observed increased lamellocyte numbers, nor melanotic masses in *Hml^P2A^>rpr* or *Hml^P2A^>Bax* larvae (*n*>20) (Arefin et al., 2015; Defaye et al., 2009).

To follow the dynamics of hemocyte ablation *in vivo* we combined *Hml^P2A^>rpr* with *srp*^Hemo^>mCherry. The ablation pattern correlated with expression analysis (Fig. 2D). Few *srp*^Hemo^+ hemocytes were still detected by the end of first larval instar [24 h after larval hatching (ALH)], but from late second instar onwards (48 h ALH) we only detected cell remnants. Hemocytes associate with multiple tissues (Cox et al., 2021; Gyoergy et al., 2018; Ayyaz et al., 2015). To address the ablation of tissue-resident hemocytes we dissociated WS-larvae and assessed cells by flow cytometry (Fig. 2E). *srp*^Hemo^+ hemocytes comprised ∼5% of live single cells in controls, but were eliminated in *Hml^P2A^>rpr* larvae. Together, this shows that *Hml^P2A^* allows almost complete ablation of plasmatocytes and crystal cells.

### Hemocyte ablation with *Hml*^P2A^-GAL4 is pupal lethal under germ-free conditions

**Fig. 3.**
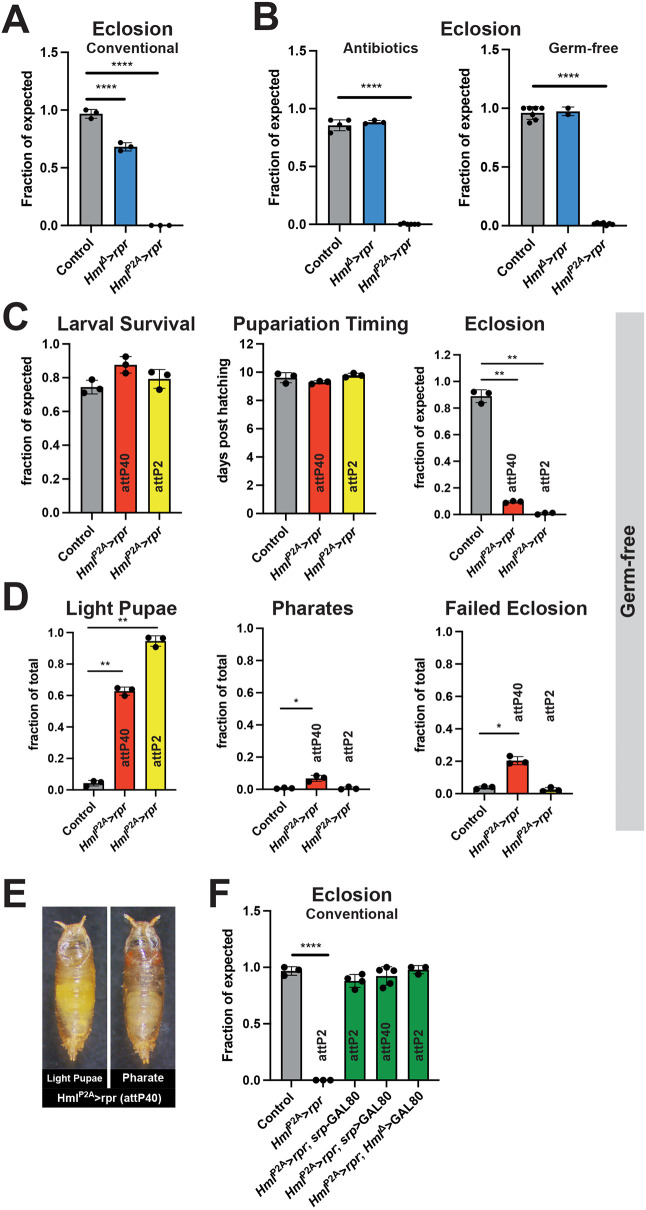
**Hemocyte ablation with *Hml*^P2A^-GAL4 is pupal lethal under germ-free conditions.** (A,B) Eclosion rates of *Hml*^Δ^>*rpr* and *Hml*^P2A^>*rpr* (attP2) animals reared at controlled density under conventional conditions (A) or on food containing 5 mg/ml ampicillin and 5 mg/ml kanamycin or under GF conditions (B). Eclosion rates were scored as number of adults obtained from pupae that were formed per vial. Each dot represents an individual vial. One-way ANOVA. (C,D) Comparison of *Hml*^P2A^-GAL4 driver inserted in attP40 or attP2 based on controlled density cultures raised under GF conditions. (C) Larval survival was scored as pupae obtained from inoculated first instar larvae. Eclosion rates were scored as number of adults obtained from pupae that were formed. Pupariation timing was scored as average over the day of pupariation for each pupae in one vial. (D) Pupal lethality was scored by determining the fraction of all pupae in a vial that terminated development before pupal stage P8 (light pupae), during stage P8-P14 (pharates) or in P15 (failed eclosion). Each dot represents an independent experiment including four vials each (C,D). One-way ANOVA. (E) Representative images of dead pupae from *Hml*^P2A^>*rpr* (attP40) used to classify light pupae (before pupal stage P8) or pharate adult stage in D. (F) Genetic rescue experiments with controlled density cultures raised under conventional conditions. *Hml*^P2A^>*rpr* (attP2) was rescued by *srp*^Hemo^-GAL80, *Hml*^P2A^>*rpr* (attP40) by *srp*^Hemo^>GAL80 or *Hml*^Δ^>GAL80. Eclosion rates were scored as number of adults obtained from pupae formed per vial, each dot represents one vial. One-way ANOVA. Controls: attP2>*rpr* (A,B,F), yw>*rpr* (C,D). Data are mean±s.d. *P<0.03; **P<0.002; ****P<0.0001.

Previous *Hml*^Δ^-GAL4 ablation studies showed a reduction in eclosion rates that were rescued by antibiotic treatment or GF conditions (Arefin et al., 2015; Charroux and Royet, 2009; Defaye et al., 2009; Shia et al., 2009). Given the improved ablation rate of hemocytes using *Hml*^P2A^-GAL4, we revisited this observation (Fig. 3; Fig. S4). First, we compared survival rates between *Hml*^P2A^-GAL4- and *Hml*^Δ^-GAL4-driven hemocyte ablation. Eclosion rates of *Hml*^Δ^>*rpr* and *Hml*^Δ^>*Bax* pupae were reduced by ∼25%; this reduction was less than previously reported (Arefin et al., 2015; Charroux and Royet, 2009; Defaye et al., 2009; Shia et al., 2009), but still statistically significant (Fig. 3A; Fig. S4B). Strikingly, eclosion rates of *Hml*^P2A^*>rpr* and *Hml*^P2A^*>Bax* pupae dropped to <5% (Fig. 3A; Fig. S4B). We then reared larvae with antibiotics (5 mg/ml ampicillin, 5 mg/ml kanamycin) or under GF conditions as previously described (Charroux and Royet, 2009; Defaye et al., 2009). Eclosion rates of *Hml*^Δ^>*rpr* and *Hml*^Δ^>*Bax* pupae were restored to control levels (Fig. 3B; Fig. S4B). In contrast, *Hml*^P2A^*>rpr* and *Hml*^P2A^*>Bax* pupae did not eclose (Fig. 3B; Fig. S4B). This pointed to an essential role for hemocytes during pupal development, independent of control of the microbiota.

Next, we analyzed lethality in more detail comparing both *Hml*^P2A^ driver lines (Fig. 3C-E; Fig. S4C,D). Larval survival was not affected under GF and conventional conditions (Fig. 3C; Fig. S4C). Larval development was extended for all genotypes under GF conditions but, relative to controls, pupariation of *Hml*^P2A^*>rpr* was only slightly delayed under conventional conditions (Fig. 3C; Fig. S4C). Eclosion rates were ∼10% for *Hml*^P2A^*>rpr* (attP40) and <1% for *Hml*^P2A^*>rpr* (attP2) (Fig. 3D). The eclosion rate for *Hml*^P2A^*>rpr* (attP40) was reduced to ∼2% under conventional conditions (Fig. S4C). To further characterize pupal lethality we scored the end-point of pupal development into three categories (Bainbridge and Bownes, 1981); arrest before pupal stage P8 (light pupae, Fig. 3D,E), between P8-P14 (pharate, Fig. 3D,E) or during P15 (failed eclosion, Fig. 3D). The majority of ‘hemoless’ pupae died before P8, with no visible eye coloration. For *Hml*^P2A^*>rpr* (attP2) this was almost 100% and not affected by GF conditions. For *Hml*^P2A^*>rpr* (attP40), GF conditions reduced the fraction of pupae that died during pharate stages (P8-14, Fig. 3D,E) and increased the number of failed eclosions and adults. This suggested that GF conditions primarily affected late pupal development. All surviving adults showed a non-inflated wing phenotype and we found residual hemocytes in escapers from *Hml*^P2A^>*rpr; srp*^Hemo^>mCherry (Fig. S4D). Together, this indicated that *Hml*^P2A^*>rpr* (attP2) causes a slightly stronger, more penetrant phenotype, which we further analyzed. *Hml*^P2A^*>rpr* (attP2) pupae showed defects during pupariation (non-retracted mouth hooks, non-everted anterior spiracles) and by 24 h post-pupariation 95% of pupae (*n*=101) had a large posterior gas bubble (Fig. S4E). This likely reflects failure to complete stage P4 (ii), the ‘moving bubble’ stage (Bainbridge and Bownes, 1981). In summary our data suggests, that ‘hemoless’ pupae die during early metamorphosis.

### Eclosion rates are rescued with hemocyte-specific expression of GAL80

In order to minimize the chance that pupal lethality in our ablation experiments was caused by off-target expression of *Hml*^P2A^-GAL4, we performed genetic rescue experiments with hemocyte-specific expression of the GAL4 inhibitor, GAL80. Eclosion rates of *Hml*^P2A^>*rpr* (attP2) were rescued by *Hml*^Δ^-QF2 driving QUAS-GAL80 (*Hml*^Δ^>GAL80), showing that *Hml*^P2A^-GAL4 has no crucial off-target expression compared with the original *Hml* driver (Fig. 3F; Fig. S4F). We then used *srp*^Hemo^, either directly driving GAL80 (*srp*^Hemo^-GAL80) or via *srp*^Hemo^-QF2 (*srp*^Hemo^>GAL80) to rescue *Hml*^P2A^>*rpr* (attP2) or *Hml*^P2A^>*rpr* (attP40), respectively (Fig. 3F; Fig. S4F). Based on the observation that *srp*^Hemo^ and *Hml*^P2A^ expression exclusively overlap in hemocytes (Fig. 1), this indicates that *Hml*^P2A^>*rpr* lethality is caused by hemocyte ablation.

### Hemocyte ablation leads to dysregulation of midgut-expressed genes

**Fig. 4.**
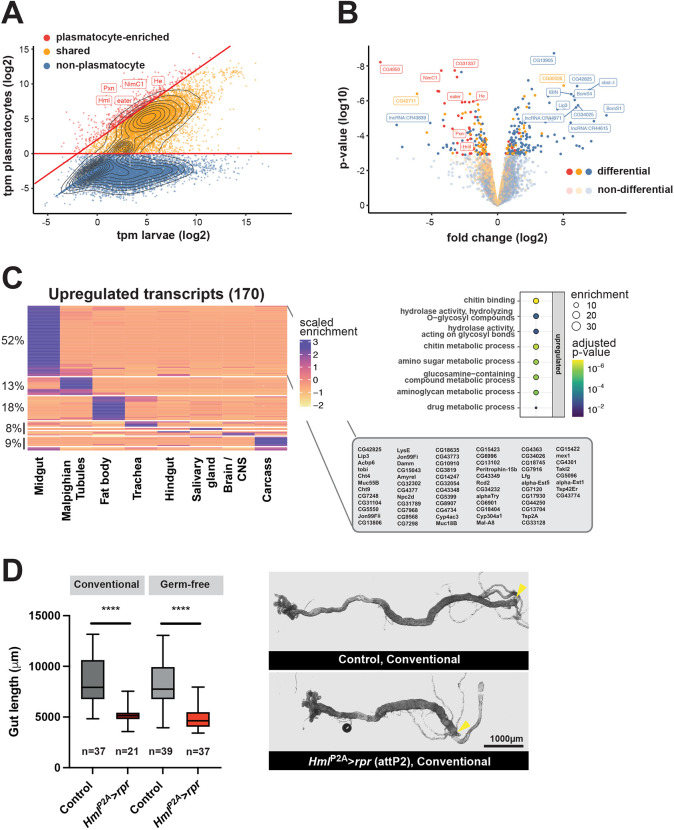
**RNA-seq analysis of ‘hemoless’ WS-larvae.** (A) RNA-seq expression analysis comparing relative expression strength (in tags per million; tpm) of transcripts in whole larvae with expression in larval plasmatocytes (both WS-larvae). Dots represent individual transcripts with overlaid density plot. Genes were classified as non-plasmatocyte (no or marginal expression in plasmatocytes; blue), shared (yellow) or plasmatocyte-enriched (>4 fold elevated in plasmatocytes; red). Hemocyte-specific transcripts are labeled. (B) Volcano plot illustrating differential transcriptome analysis of *Hml^P2A^*>*rpr* versus *attP2*>*rpr* WS-larvae. Dots mark log2 fold changes and log10 differential expression *P*-values for individual genes. Genes are colored by assignment as in A, with non-significantly regulated transcripts in lighter colors. The 15 most significantly regulated transcripts and hemocyte-specific transcripts are labeled. (C) Heatmap showing scaled tissue enrichment derived from FlyAtlas2 for upregulated protein coding transcripts. The fraction of transcripts within each k-means cluster is indicated in percent, the tissue type below the heatmap. A list of midgut-specific transcripts that were upregulated in response to hemocyte ablation is shown. Gene ontology terms enriched within upregulated transcripts, *P*-values and enrichment effect sizes are indicated. (D) Midgut lengths from female *Hml*^P2A^>*rpr* (attP2) versus yw>*rpr* (Control) WS-larvae reared under conventional or GF conditions and dissected in Schneider's media (containing Ca^2+^). Midgut length was determined from the anterior of whole guts (left) to the attachment site of Malpighian tubules that mark transition to the hindgut (yellow arrowhead). Number of guts are indicated. One-way ANOVA. Box plot shows median values (middle bars) and first to third interquartile ranges (boxes); whiskers indicate minimum and maximum values. ****P<0.0001.

To analyze the consequences of hemocyte ablation before lethality in early pupal stages, we generated RNA-seq datasets for whole WS-larvae and isolated plasmatocytes and classified transcripts as: (1) not expressed in plasmatocytes, (2) similarly present in both datasets (shared) or (3) enriched in plasmatocytes (Fig. 4A). To address potential systemic responses we further analyzed tissue-specific enrichment of transcripts based on data available at FlyAtlas2 (Leader et al., 2018) (Fig. S5B). Plasmatocyte-enriched transcripts were detected to a variable degree in multiple tissues (Fig. S5B), reflecting either low-level expression outside plasmatocytes or the presence of plasmatocytes in these tissues (Ayyaz et al., 2015; Cattenoz et al., 2020; Cox et al., 2021). Known hemocyte-specific transcripts (*Hml*, *He*, *eater*, *Pxn* and *NimC1*) were detected as plasmatocyte-enriched (Fig. 4A) and present in the larval carcass (Fig. S5B), likely due to the association of sessile hemocytes with the cuticle or lymph glands in the carcass. These transcripts were also significantly depleted in the differential expression analysis between *Hml^P2A^>rpr* and control larvae (Fig. 4B; Table S1).

We could not identify a tissue-specific response for downregulated transcripts, as only a minority of them were non-plasmatocyte (46/131, Fig. S5C) and Gene Ontology (GO) analysis revealed phagocytosis as the only significantly enriched process or function. In contrast, upregulated transcripts primarily comprised non-plasmatocyte transcripts (131/170) and a majority of them was expressed in the midgut (Fig. 4C; Fig. S5C). GO term analysis showed a strong enrichment of genes associated with chitin metabolism (Fig. 4C). To further analyze phenotypic differences in larval gut morphology, we dissected guts from WS-larvae and analyzed them using electron microscopy (EM) and fluorescent microscopy. Larval gut lengths were significantly decreased in *Hml^P2A^>rpr* compared with control (Fig. 4D; Fig. S6A,B). However, we did not observe a striking difference in gut architecture by either EM or fluorescent microscopy (Fig. S6C,D). Dissected *Hml^P2A^>rpr* pupal guts 24 h post-pupariation resembled control guts from 12 h post-pupariation, which is approximately the time point at which *Hml^P2A^>rpr* lethality starts (stage P4, Fig. S6E).

Taken together, we have shown an essential role for hemocytes in post-embryonic development beyond the control of microorganisms. Hemocyte ablation in our model starts in early larval development and appears to be complete by mid-larval stages. The main advantages of our model compared with previous systems are improved transgene expression over *Hml*^Δ^ and higher specificity for hemocytes compared with *srp*^Hemo^. In contrast to previous studies, we achieved depletion of hemocytes to <1% of control animals, which leads to pupal lethality that can no longer be rescued by GF conditions. One of these studies showed that hemocyte-derived signaling promotes basal and damage- or infection-induced proliferation of intestinal stem cells in adults but failed to detect a larval gut phenotype after ablation of hemocytes with *Hml*^Δ^ (Ayyaz et al., 2015). The striking reduction in larval midgut length that we observed could indicate a similar developmental function of hemocytes, which was previously obscured by incomplete hemocyte ablation. However, we cannot exclude that lethality resulted from pleiotropic defects that we observed later in development.

A drawback of apoptosis-driven depletion of hemocytes is that the increase in apoptotic bodies may in itself induce a physiological response in the fly, including upregulation of immune-related genes (Arefin et al., 2015). In future studies it would be interesting to assess whether the genetic manipulation of specific hemocyte functions recapitulates pupal lethality, as seen when hemocyte migration is impaired in embryos (Matsubayashi et al., 2017).

## MATERIALS AND METHODS

### *Drosophila melanogaster* strains

Fly strains obtained from Bloomington Drosophila Stock Center were *Hml*^Δ^*-*GAL4 (30139), *UAS-2xEGFP* (6874), *UAS-rpr* II (5824), *UAS-rpr* X (5823), *srp*Hemo*-*QF2 (78365), *srp*Hemo*-*GAL80 (78366), *Hml*^Δ^*-*QF2 (66468), *QUAS-*GAL80 (51950), *QUAS-*mCherry (5227) and attP2 (8622). The *P{UAS-Bax.G}* integration on chromosome II (*UAS-Bax*) was a gift from Carla Saleh (Department of Virology, Institute Pasteur, Paris, France) and balanced with CyO, *P{ActGFP}JMR1*. The *Hml*^P2A^-GAL4 lines were constructed in this study. Genotypes derived from these strains are listed in Table S2.

### Generation of *Hml*^P2A^*-*GAL4 Flies

A 3477 bp fragment was synthesized (Eurofins) containing 840 bp upstream of the *Hml* transcription start site and the *Hml* transcript up to the end of exon 9 (bp13845367-bp13848766, dm6), directly followed by an *Avr*II site, a P2A translation skip sequence and a *Xba*I site. The *Xba*I site was used to insert the GAL4 coding sequence from pGawB followed by a SV40 3′ untranslated region from pUASt. The construct was assembled in a backbone derived from pDESTR3R4-φ*C31att*B (Gunesdogan et al., 2010), containing a *w*^+mc^ transformation marker and attB integration sequence. Transgenic lines were generated in *P{CaryP}attP40* and *P{CaryP}attP2* by Rainbow Transgenics. The resulting *P{Hml-GAL4.P2A}attP40* and *P{Hml-GAL4.P2A}attP2* integrations were crossed out to remove integrase transgenes before further use.

### Controlled density cultures

Cultures were raised on rich cornmeal molasses food (25 g Agar-Agar, 14.4 g yeast, 8 g soy flour, 64 g yellow cornmeal, 64 g light malt extract, 17.6 g molasses, 6 ml propionic acid, 0.8 g Nipagin/l). For GF cultures, medium was autoclaved before adding propionic acid. For antibiotic treatment, 5 mg/ml ampicillin (Sigma-Aldrich) +5 mg/ml kanamycin (Sigma-Aldrich) was added before pouring the food at ∼50°C. For controlled density cultures, embryos were collected on apple juice agar plates at 25°C in a 12 h light/dark cycling incubator. First instar larvae were picked from plates and seeded at 100 or 120 larvae per vial. Conventional/antibiotics cultures contained live yeast. For staged cultures, first instar larvae that hatched within a 5 h time window were seeded. Larvae were raised at 25°C in a 12 h light/dark cycling incubator.

### Larval/pupal survival assays

For crosses involving *P{UAS-rpr.C}14*, larval survival was scored as fraction of pupae formed from seeded first instar larvae. For crosses involving *P{UAS-Bax.G}*, larval survival was scored relative to internal control genotype (*Hml*^P2A^*>Bax* per *Hml*^P2A^*>Bal*). For crosses involving *P{UAS-rpr.C}14*, pupal survival was scored as fraction of pupae that developed to adults (or a specific pupal stage) from all pupae that were formed in a vial. For crosses involving *P{UAS-Bax.G}*, pupal survival was scored as number of adults relative to internal control genotype (*Hml*^P2A^*>Bax per Hml*^P2A^*>Bal*). From this cross we expected 1:1 *Hml*^P2A^*>Bax*:*Hml*^P2A^*>Bal* adults if there was no effect in *Bax*-expressing animals. The fraction of expected therefore was scored relative to balancer carrying animals. As balancer chromosomes can, to some degree, reduce fitness, the fraction of expected can be higher than one in some cases. For analysis of pupariation timing, newly formed pupae were scored in 24 h intervals. Vials were left 1 week longer than eclosion was observed in control genotypes to check for late eclosing adults. All pupae that did not eclose were inspected and scored as light pupae (<stage P8), pharates (stage P8-P14) or failed eclosion (stage P15).

### Germ-free cultures

Embryos were collected from crosses for 6 h at 25°C. Embryos were washed twice in a 100 µm cell-strainer with PBTx.01 [PBS, 0.01% Triton X-100 (Sigma-Aldrich)] and transferred to 70% ethanol in a clean bench. After 5 min, embryos were dechorionated in 50:50 Clorax/water (2.5% HOCl final) for 2 min and washed three times in sterile PBTx.01. For initial experiments (Fig. 2B; Fig. S4B) embryos were suspended in PBTx.01 and total number of embryos approximated by counting from an aliquot. An average of 100 embryos were seeded in sterile food vials by pipetting the respective amount of embryo suspension. For all other experiments, embryos were transferred to sterile agar plates and hatched larvae were picked for a time window of 5 h using a Lynx EVO stereomicroscope (Vision Engineering) in a clean bench. To check for GF conditions, five adults/pupae from each culture were homogenized in 100 µl PBS and plated on YPD. Plates were sealed and incubated for 3 days at 30°C. Plates from conventional conditions that were included with each plating showed a lawn of yeast/bacteria; GF cultures that showed any growth on plates were excluded from the analysis (<5% of cultures).

### Hemocyte extraction by bleeding

Larvae were collected and extensively washed with water in 100 µm cell-strainers to remove debris followed by 5 min in 70% ethanol. Sessile hemocytes were dislodged by extensively rubbing the larvae with a paint brush. Larval cuticles were ripped open from posterior to anterior using fine forceps and hemocytes bled out.

### Hemocyte quantification and staining

For hemocytometer counts, larvae were bled into 20 µl PBS, 5 mM EDTA, 1:250 protease inhibitor cocktail (Sigma-Aldrich) on parafilm. For antibody staining, five larvae were bled into 200 µl Schneider's medium (Sigma-Aldrich) with 10 mM N-Acetyl-L-Cysteine (Sigma-Aldrich) in eight-well µ-slide dishes (ibidi). Cells were allowed to adhere for 30 min, washed twice with Schneider's medium and fixed for 10 min with 4% paraformaldehyde (Sigma-Aldrich), 50 mM EDTA in PBS. Samples were washed with PBTx (PBS, 0.1% Triton X-100) and blocked for 30 min with 10% goat serum (Sigma-Aldrich) in PBTx (PBTx-GS). Primary antibodies 1:60 in PBTx-GS (anti-Nimrod C1 clones P1a/P1b, anti-Hemese clone H2, anti-Attila clones L1a/L1b/L1c, gift from Istvan Ando, Szeged, Hungary; Kurucz et al., 2007b) were incubated overnight at 4°C. After three washes with PBTx, samples were incubated with AlexaFluor 647 goat anti mouse (1:500, Abcam, ab150115) and 0.1 mg/ml RNase A (Sigma-Aldrich) in in PBTx-GS for 2 h at room temperature. After two washes with PBTx, samples were overlaid with 5 µM DAPI (Roth) in PBTx. Entire wells were scanned using a Leica SP8 confocal microscope and images analyzed in Fiji. For quantification of ablation experiments, images were first segmented based on antibody staining. Within antibody-positive area, segmentation based on DNA staining with particle limits 5-50 µm^2^ was used to determine cell numbers. Lamellocytes were selected manually from scans of entire wells stained with anti-Hemese antibody and checked for a minimum single cell size of 300 µm^2^, which was ∼2.5 times larger than the average size of Hemese-positive cells (∼120 µm^2^). For quantification of co-expression, images were segmented based on DNA staining with a lower area limit of 20 µm^2^ and signal thresholds were adjustment to yield an average area of 55 µm^2^ over all detections. Analysis was then limited to particle sizes between 28-83 µm^2^. Within these regions, average signal intensities for EGFP, mCherry and Alexa 647 were measured. Thresholds for positive detections were set in R based on the analysis of signal histograms. For crystal cell quantification, larvae were picked and washed in PBS and then heat-shocked at 65°C for 10 min, which causes the crystal cells to melanize and turn black. For each larva, a dorsal and ventral image was taken using a Leica M205 stereomicroscope. Crystal cells were counted manually.

### Hemocyte flow cytometry

For analysis of EGFP expression, 50 larvae were bled into the lid of a 1.5 ml Eppendorf tube containing 200 µl Schneider's medium, 1:250 protease inhibitor cocktail (Sigma-Aldrich). Carcasses were removed and the cell solution was transferred to a 1.5 ml Eppendorf tube containing 800 µl fresh Schneider's medium and passed through a 70 µm Flowmi tip filter (Sigma-Aldrich). Cells were analyzed on a MACSQuant Analyser (Miltenyi Biotec). For the analysis of tissue-associated hemocytes, 20 larvae were washed in water and homogenized in 1 ml of PBS with 20-30 strokes in a 1 ml loose fit Dounce. The suspensions were passed through a 40 µM cell-strainer by centrifugation into Schneider's medium containing 25% fetal calf serum (FCS), 2 mM EDTA at 1500 ***g*** for 10 min. The supernatant was discarded and the pellet resuspended in 1 ml Schneider's medium and analyzed using CytoFLEX (Beckman). Data were analyzed using FlowJo software.

### Live imaging experiments

Imaging of embryos and larvae was performed in Frame-Seal Hybridization Slide Chambers (15×15 mm, BioRad) filled with a gel of 30% (w/v) OptiPrep (Sigma-Aldrich) and 30% (w/v) Pluronic F-127 (Sigma-Aldrich) in PBS on a glass slide closed with a coverslip. The gel was kept liquid at 4°C and solidified at room temperature. Embryos were dechorionated in 50:50 Clorax:water (2.5% HOCl final) for 3 min, washed with embryo saline (0.7% NaCl, 0.01% Triton X-100) and glued to the coverslip that sealed the chamber. Larvae were washed in embryo saline, anesthetized with ether according to published protocol (Kakanj et al., 2020) and glued to the slide side of the chamber. Adult flies were anesthetized with ether and glued to a glass slide. Imaging of adults and whole larvae was carried out using a Leica M205 stereomicroscope, embryos and larval details were imaged on a Leica SP8 confocal microscope.

### Larval and pupal gut analysis

Larvae were washed in water and transferred to either Schneider's medium or PBS for dissection. Intact guts were allowed to contract for 30 min and fixed for 10 min in 4% paraformaldehyde, 50 mM EDTA in PBS. For determination of gut length, guts were mounted in fixative in Frame-Seal Hybridization Slide Chambers, imaged with a Leica M205 stereomicroscope and analyzed in Fiji. For fluorescent staining, guts were washed in PBTx and stained in PBTx, 1 mg/ml RNase A, 5 µM DAPI, 300 nM Alexa Fluor 647 Phalloidin (Thermo Fisher Scientific) for 30 min. Samples were mounted in Frame-Seal Hybridization Slide Chambers and imaged with a Leica SP8 confocal microscope. For electron microscopy, midguts were post-fixed with 2.5% glutaraldehyde (Electron Microscopy Sciences) and embedded in groups into small cubes of low melting agarose. These were then further post-fixed with 0.5% osmium-tetroxide and tannic acid, contrasted with uranyl-acetate, dehydrated in a graded ethanol series and infiltrated in Polybed (Polysciences). The cubes were placed in flat embedding molds with Polybed. After polymerization, the blocks were trimmed to the desired sectioning plane and sections were cut at 60 nm. Specimen were analyzed in a Zeiss LEO 906E transmission electron microscope, equipped with a side-mounted digital camera (Morada, SIS-Olympus), at 100 kV.

### Plasmatocyte RNA-seq

OreR larvae were raised at controlled density as described above. Plasmatocytes were extracted as described above into complete media in tissue-culture-treated dishes (Schneider's medium with 10% FCS and 10 mM N-acetyl-L-cysteine). For each sample, 80 larvae were bled. The larval carcasses were then removed and the plasmatocytes were allowed to attach for 10-15 min. Afterwards, plasmatocytes were washed four times with PBS and lysed in 900 µl TRIzol. Samples were moved to fresh prespun phase lock heavy tubes (5PRIME) and 250 µl chloroform was added to each sample, mixed thoroughly and centrifuged (12,000 ***g***, room temperature, 15 min). The upper aqueous phase was moved to a fresh DNA LoBind tube and mixed with 550 µl isopropanol and 1 µl glycogen (20 mg/ml, RNAse free). Samples were mixed by inverting and then incubated for 30 min in the freezer at −20°C. Samples were centrifuged (16,000 ***g***, 4°C, 10 min) and the supernatant was removed carefully without disrupting the pellet. The pellet was resuspended in 100 µl ultra-pure water with 300 mM sodium acetate and 1 µl glycogen (20 mg/ml, RNAse free). Then 300 µl ethanol was added and the sample was incubated for 20 min at −20°C, centrifuged (16,000 ***g***, 4°C, 10 min) and the supernatant discarded. The pellet was washed twice by adding 1 ml 70% ethanol (prepared with ultra-pure water), each time spinning down the pellet (16,000 ***g***, 4°C, 3 min). Afterwards, all supernatant was drained and the pellet was dried until no liquid was visible. The pellet was then resuspended in 15 µl of ultra-pure water and stored at −80°C. Libraries from samples were generated at the Max Planck Genome Centre in Cologne, Germany, using the New England Biolabs Next Ultra II Directional RNA library kit with polyA mRNA enrichment from ∼100 ng total RNA. Libraries were pooled and sequenced to a minimum of 16 million uniquely mapped single end 150 bp reads per sample at the Max Planck Genome Centre.

### Larval RNA-seq

*Hml*^P2A^-GAL4 in attP2 or control *P{CaryP}attP2* males were crossed to *UAS-rpr* X virgins and larvae were raised as described above. Ten male WS-larvae were collected per sample across independent replicates and snap-frozen in liquid nitrogen. Larvae were transferred to Lysing Matrix E homogenization tubes with 1 ml of TRIzol and ruptured on high settings in a FastPrep tissue homogenizer (MP Biomedicals). The supernatant was transferred to a fresh tube and spun down for 2 min at maximum speed. Then 800 µl of the TRIzol sample was transferred to a fresh prespun phase lock heavy tube and 200 µl chloroform was added. Phases were separated by spinning at 12,000 ***g*** for 15 min at 4°C. The upper aqueous phase was transferred to a fresh tube and mixed with 500 µl isopropanol. The resulting mix was spun at 20,000 ***g*** for 15 min at 4°C. All supernatant was drained and the pellet resuspended in 30 µl DNAse solution (Ambicon, final concentration 0.2 U/µl) and incubated for 1 h at 37°C. RNA was purified using the RNeasy Plus kit (Qiagen) by adding 270 µl RTL buffer and isolated according the manufacturer's instructions. RNA concentration was determined by Nanodrop and integrity was checked by Bioanalyzer. Libraries from samples were generated at the Max Planck Genome Centre using the New England Biolabs Next Ultra II Directional RNA library kit with polyA mRNA enrichment from ∼100 ng total RNA. Libraries were pooled and sequenced to a minimum of 10 million uniquely mapped 2×75 bp paired end reads per sample at the Max Planck Genome Centre.

### RNA data mapping and analysis

The reference genome fasta sequence file of the Berkeley *Drosophila* Genome Project assembly dm6 and the related gtf genome annotation file for dm6 of ensembl release 91 (dm6.91) were downloaded from ensembl (www.ensmbl.org) (Zerbino et al., 2018). A reference genome index was generated using dm6.91 using STAR-2.7.0e (Dobin et al., 2013) and used to map the fastq files. Quality control of RNA-seq mapping was performed using RSeQC (Zhang et al., 2021). All quality control files for FastQC, STAR mapping and RSeQC were aggregated and visualized using MultiQC (Ewels et al., 2016) and all data were checked to make sure the library and sequencing was of good quality. Once a dataset passed quality control, the gene level read counts were determined from bam files using the subread package (Liao et al., 2013). The gene level read counts were then loaded into R. For principal component analysis (PCA) the matrix of gene level read counts was transformed using the DESeq2 (Love et al., 2014) rlog function, from which the 1000 most-variant genes were selected. PCA was performed using the stats package prcomp function. For differential expression analysis, gene level read counts were processed using the edgeR package (Robinson et al., 2010) with the quasi-likelihood general linear model approach according to the manual. GO Term enrichment was performed using GOrilla (http://cbl-gorilla.cs.technion.ac.il/) (Eden et al., 2009), testing enrichment of regulated protein coding gene sets against all genes detected in the experiment.

### Tissue enrichment analysis

All available datasets for protein coding genes detected in the RNA-seq experiments were downloaded as text files from the web interface of FlyAtlas2 (http://flyatlas.gla.ac.uk/FlyAtlas2/index.html?page=home#). Tissue enrichments of individual transcripts were extracted from these files, leaving out the enrichment in Garland cells as no documentation was available on how these cells were purified. The enrichment values were scaled in R and the resulting *z*-scores visualized as heatmaps after k-means clustering.

## Supplementary Material

10.1242/develop.200286_sup1 Supplementary information
