# Supplementary material for: Hemocytes are essential for Drosophila melanogaster post-embryonic development, independent of control of the microbiota
Source: Development. 2022 Sep 29;149(18):dev200286. doi: 10.1242/dev.200286 (PMC9641648; doi:10.1242/dev.200286)
Supplement: Supplementary information [file develop-149-200286-s1.pdf]

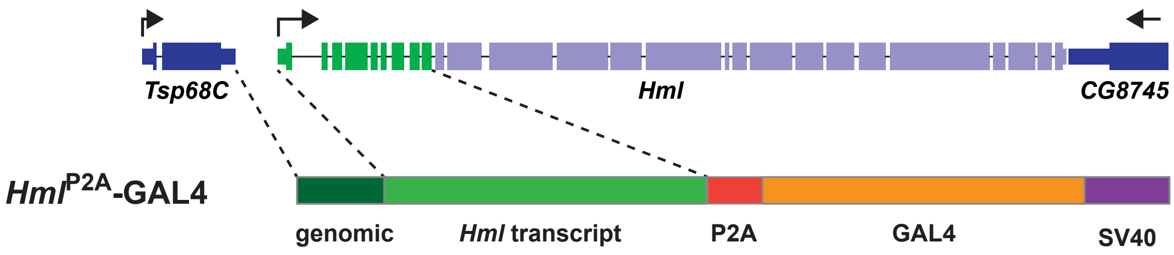

Fig. S1. Schematic of the *Hml* gene and the *Hml*^{P2A}-GAL4 construct.

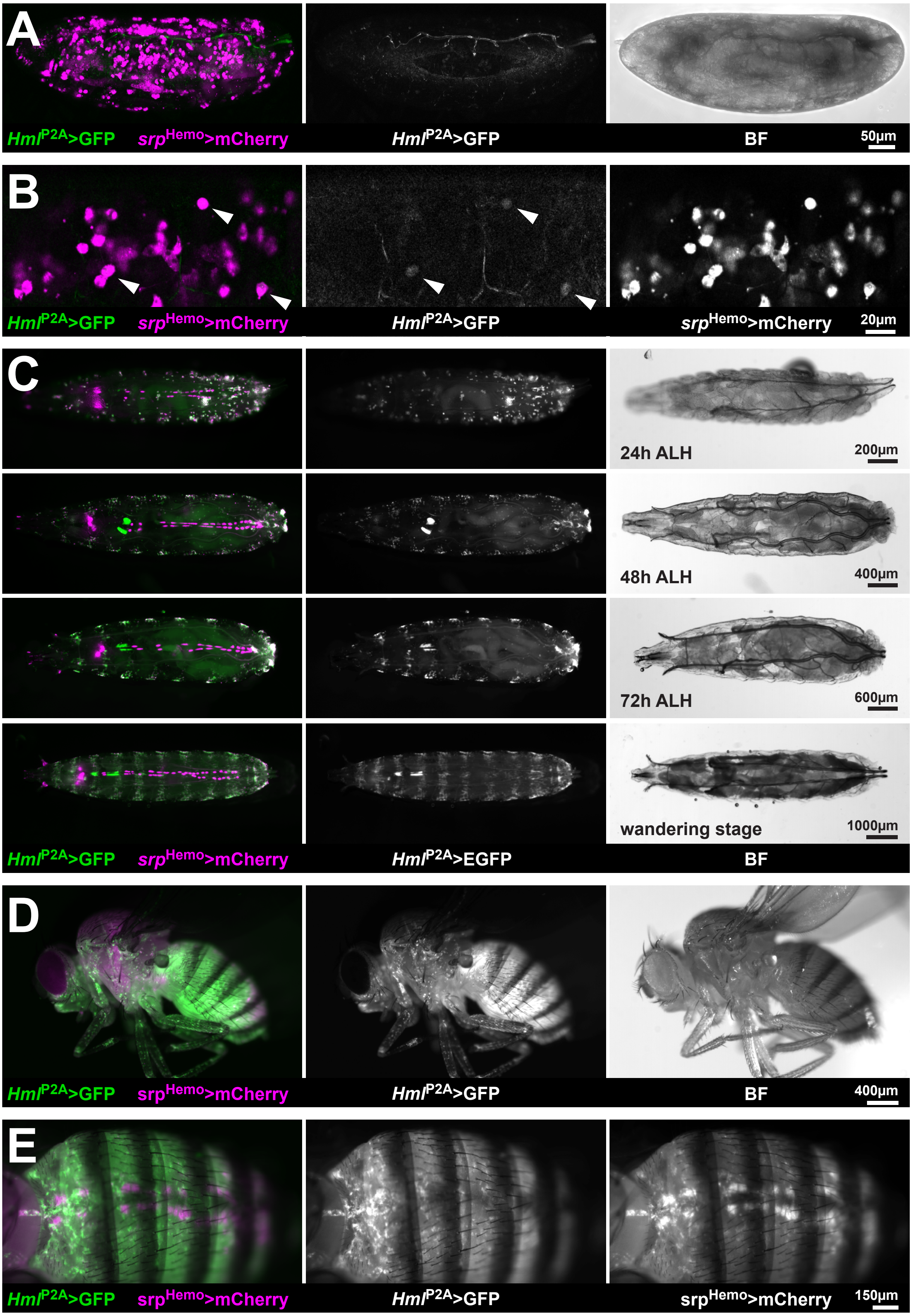

Development • Supplementary information

Fig. S2. (A-E) Fluorescent live imaging of *Hml*^{P2A}>GFP (attP40); *srp*Hemo>mCherry animals reared under conventional conditions and controlled density (B-E).

(A) Maximum projection of an image stack that included ~80µm from the apical surface of a stage 17 embryo briefly before hatching, as indicated by air filled trachea.

(B) Maximum projection of the hemocytes containing apical region of the embryo shown in (A). Arrowheads mark hemocytes with faint expression of *Hml*^{P2A}>GFP.

(C) Dorsal view of whole larvae in a time course of late first instar (24h after larval hatching, ALH), late second instar (48 h ALH), mid third instar (72 h ALH) and WS-larvae (wandering stage). BF indicates bright field images. Outside hemocytes, *srp*Hemo>mCherry is detected in some cells of unknown identity at the anterior of larva (left), in Garland cells that form a group of cells in the interior at about 20% larval length close to the brain, and in pericardial nephrocytes that form two rows of cells along the heart at the dorsal midline. We did not detect *Hml*^{P2A}>GFP expression in any of these regions.

(D & E) Whole mount female fly (D) and close up of the abdomen of a female fly showing expression of *srp*^{Hemo}>mCherry in adult pericardial nephrocytes (E).

Development • Supplementary information

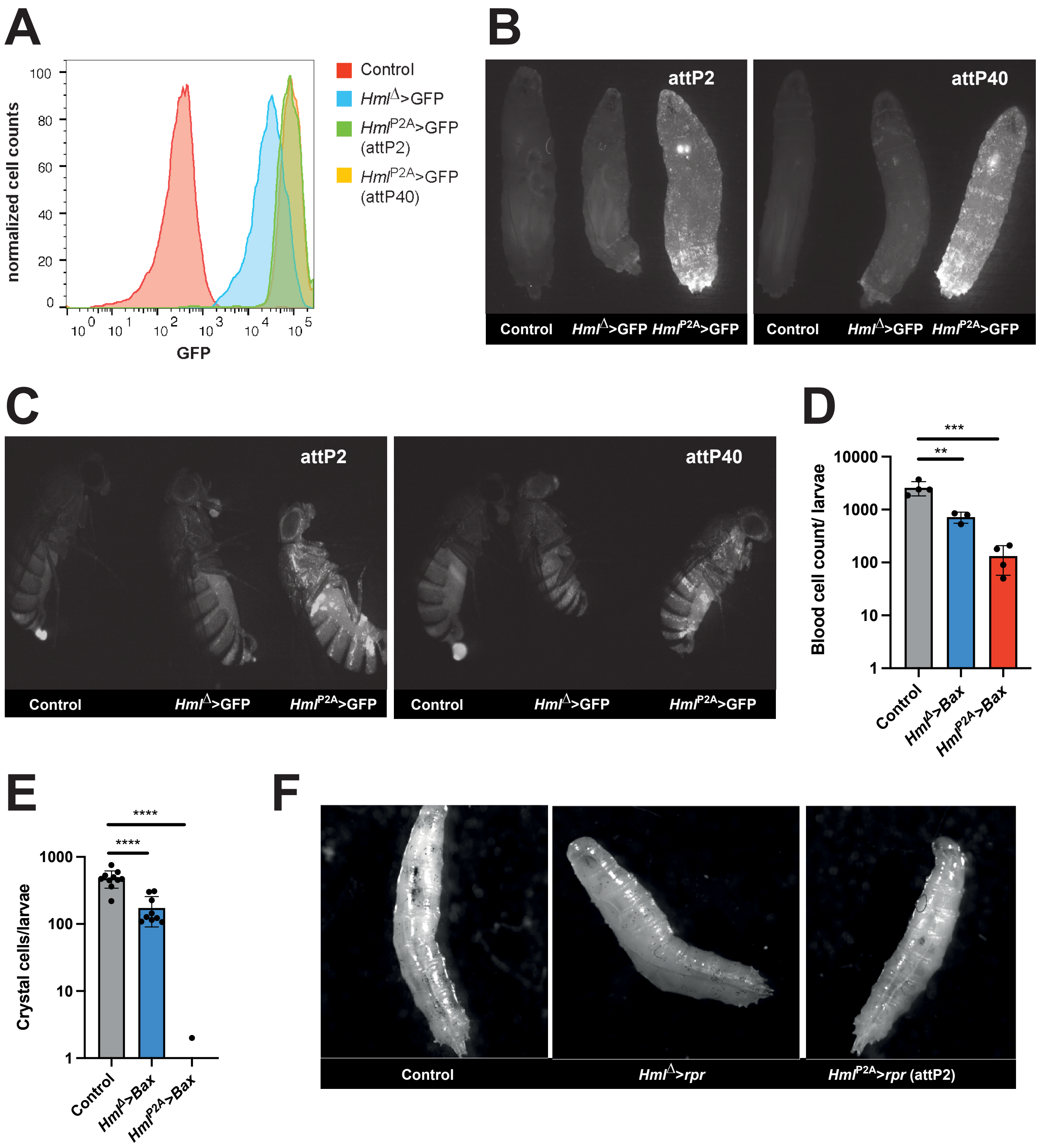

Development • Supplementary information

Fig. S3. (A) Flow cytometry analysis of extracted hemocytes from WS-larvae comparing the expression of *Hml*$^{\Delta}$>GFP, *Hml*^{P2A}>GFP (attP2) and *Hml*^{P2A}>GFP (attP40). Representative histogram from two independent experiments.

(B) Whole mount fluorescence microscopy of WS-larvae comparing the expression of *Hml*$^{\Delta}$>GFP to *Hml*^{P2A}>GFP (attP2; left) and Hml$^{\Delta}$>GFP to Hml^{P2A}>GFP (attP40; right). Representative images from two independent experiments.

(C) Whole mount fluorescence microscopy of adult females comparing the expression of *Hml*$^{\Delta}$>GFP to *Hml*^{P2A}>GFP (attP2; left) and *Hml*$^{\Delta}$>GFP to *Hml*^{P2A}>GFP (attP40; right). Representative images from two independent experiments.

(D) Blood cell counts by hemocytometer of hemocytes from WS-larvae from *Bax* mediated ablation in *Hml*$^{\Delta}$>*Bax* or *Hml*^{P2A}>*Bax* (attP2). One-way ANOVA was performed

(E) Crystal cell counts by whole mount microscopy of WS-larvae after *Bax* mediated ablation in *Hml*$^{\Delta}$>*Bax* or *Hml*^{P2A}>*Bax* (attP2). Each dot represents counts from a single animal. One-way ANOVA was performed.

(F) Representative images of *Hml*$^{\Delta}$>*rpr* or *Hml*^{P2A}>*rpr* (attP2) WS-larvae used for crystal cell quantification.

Data are mean±s.d. (D,E).

Controls: attP2>GFP (A-C), attP2>*rpr* (D & E), attP2>*rpr* (F).

** $p<0.002$; *** $p<0.0002$; **** $p<0.0001$

Development • Supplementary information

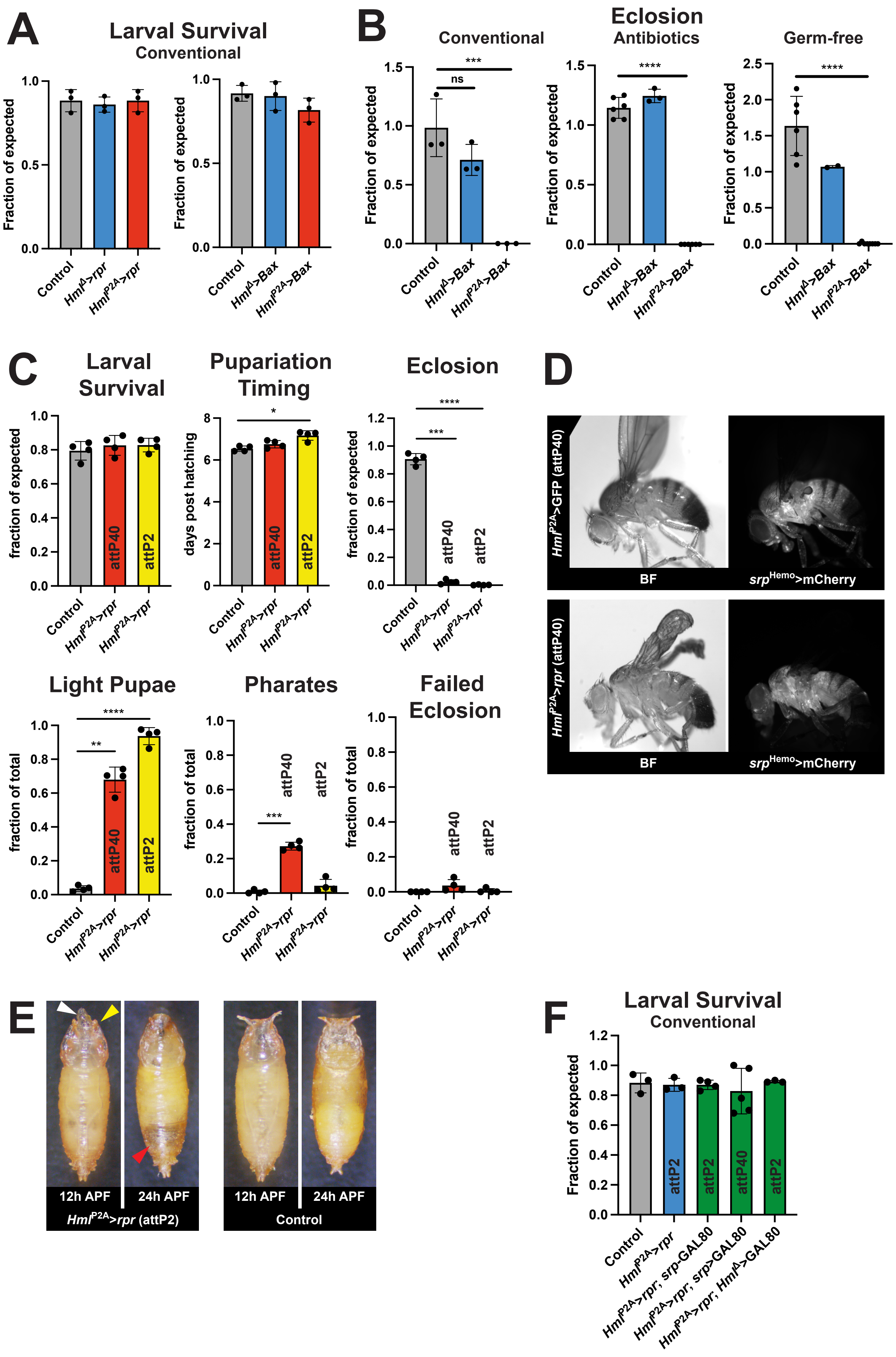
A
Larval Survival
Conventional
Fraction of expected
Control
Hml^Δ>rpr
Hml^P2A>rpr
Hml^Δ>Bax
Hml^P2A>Bax
B
Eclosion
Conventional
Antibiotics
Germ-free
Fraction of expected
ns
***
****
C
Larval Survival
Pupariation Timing
Eclosion
fraction of expected
days post hatching
attP40
attP2
Light Pupae
Pharates
Failed Eclosion
fraction of total
D
Hml^P2A>GFP (attP40)
Hml^P2A>rpr (attP40)
BF
srp^Hemo>mCherry
E
12h APF
24h APF
Hml^P2A>rpr (attP2)
Control
F
Larval Survival
Conventional
Fraction of expected
Hml^P2A>rpr, srp-GAL80
Hml^P2A>rpr, srp>GAL80
Hml^P2A>rpr, Hml^Δ>GAL80

Fig. S4. (A) Larval survival from controlled density cultures under conventional conditions. Survival was scored as pupae obtained from inoculated first instar larvae for *Hml*$^{\Delta}$*>rpr* or *Hml*^{P2A}*>rpr* (attP2). For *Hml*$^{\Delta}$*>Bax* or *Hml*^{P2A}*>Bax* (attP2) survival was scored relative to the internal controls *Hml*$^{\Delta}$*>Bal* or *Hml*^{P2A}*>Bal* as fraction of GFP negative per GFP positive pupae (see materials and methods section for details). Each dot represents an individual vial. One-way ANOVA were performed.

(B) Eclosion rates from *Hml*$^{\Delta}$*>Bax* or *Hml*^{P2A}*>Bax* (attP2) animals reared at controlled density under conventional conditions, on food containing 5 mg/mL Ampicillin and 5 mg/mL Kanamycin or under germ-free conditions. Eclosion rates were scored relative to the internal controls *Hml*$^{\Delta}$*>Bal* or *Hml*^{P2A}*>Bal* as fraction of Cy+ per Cy- adults, which can produce fractions of expected >1 (see materials and methods section for details). Each dot represents an individual vial. One-way ANOVA were performed.

(C) Comparison of *Hml*$^{\mathrm{P2A}}$-GAL4 driver inserted in attP40 or attP2 based on controlled density cultures raised under conventional conditions. Larval survival was scored as pupae obtained from inoculated first instar larvae. Eclosion rates were scored as number adults obtained from pupae that were formed and pupariation timing as average over the day of pupariation for each pupae in one vial. Pupal lethality was scored by determining the fraction of all pupae in a vial that terminated development before pupal stage P8 (light pupae), during stage P8-P14 (pharates) or in P15 (failed eclosion). Each dot represents an independent experiment including 4 vials each. One-way ANOVA were performed.

(D) Representative whole mount images of *Hml*$^{\mathrm{P2A}}$>GFP (attP40), *srp*^{Hemo}>mCherry animals (top) and surviving *Hml*$^{\mathrm{P2A}}$*>rpr* (attP40), *srp*^{Hemo}>mCherry animals. *Hml*$^{\mathrm{P2A}}$*>rpr* (attP40), *srp*Hemo>mCherry animals still contained *srp*^{Hemo}>mCherry and showed a wing inflation phenotype.

(E) Pupal phenotypes of *Hml*^{P2A}*>rpr* (attP2) animals. In early pupae (12h after pupariation formation) defective retraction of mouth hooks (white arrowhead) and lack of clearly visible anterior spiracles (yellow arrowhead) is evident. By 24 h after pupariation formation 95% of pupae show a large posterior air bubble (red arrowhead).

(F) Larval survival from controlled density cultures under conventional conditions. Survival was scored as pupae obtained from inoculated first instar larvae. Genotypes used here are identical with those in Figure 3F. One-way ANOVA were performed.

Data are mean±s.d.. Controls: attP2>*rpr* and attP2>Bax (A), attP2>Bax (B), yw>*rpr* (C & E), attP2>*rpr* (F). * p<0.03; ** p<0.002; *** p<0.0002; **** p<0.0001. ns, not significant.

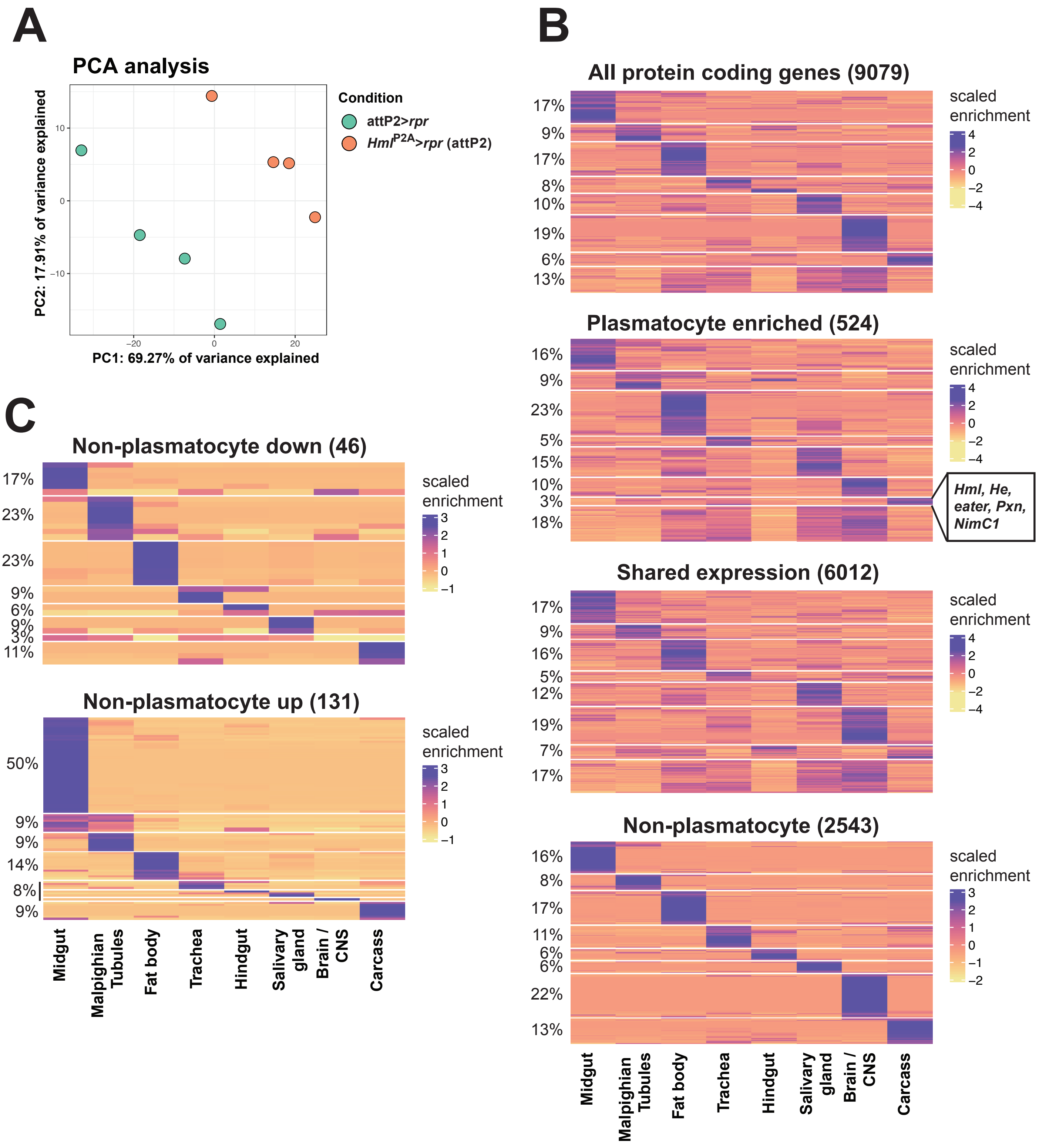

Development • Supplementary information

Fig. S5. (A) Principle component analysis (PCA) of RNAseq replicate data from *Hml*^{P2A}*>rpr* and *attP2>rpr* WS-larvae. Samples of both genotypes are separated by a shared component in PC1 and PC2, suggesting a consistent transcriptional effect between replicates.

(B) Heatmap showing scaled tissue enrichment of all protein coding transcripts detected in our differential expression analysis for which data was available at FlyAtlas2 (All protein coding genes) and for subsets identified by our RNAseq analysis as plasmatocyte-enriched, shared or non-plasmatocyte expressed with the number of transcripts in brackets. Known plasmatocyte specific transcripts, which are part of the plasmatocyte enriched subset, show tissue enrichment in the carcass. The percentage of transcripts that are in each k means cluster of the heatmaps is indicated to the left.

(C) Heatmap showing scaled tissue enrichment of protein coding transcripts that were downregulated and classified as non-plasmatocyte expressed (upper panel) or upregulated and classified as non-plasmatocyte expressed (lower panel). Transcript numbers are indicated by brackets and the percentage of transcripts that are in each k means cluster of the heatmaps is indicated to the left.

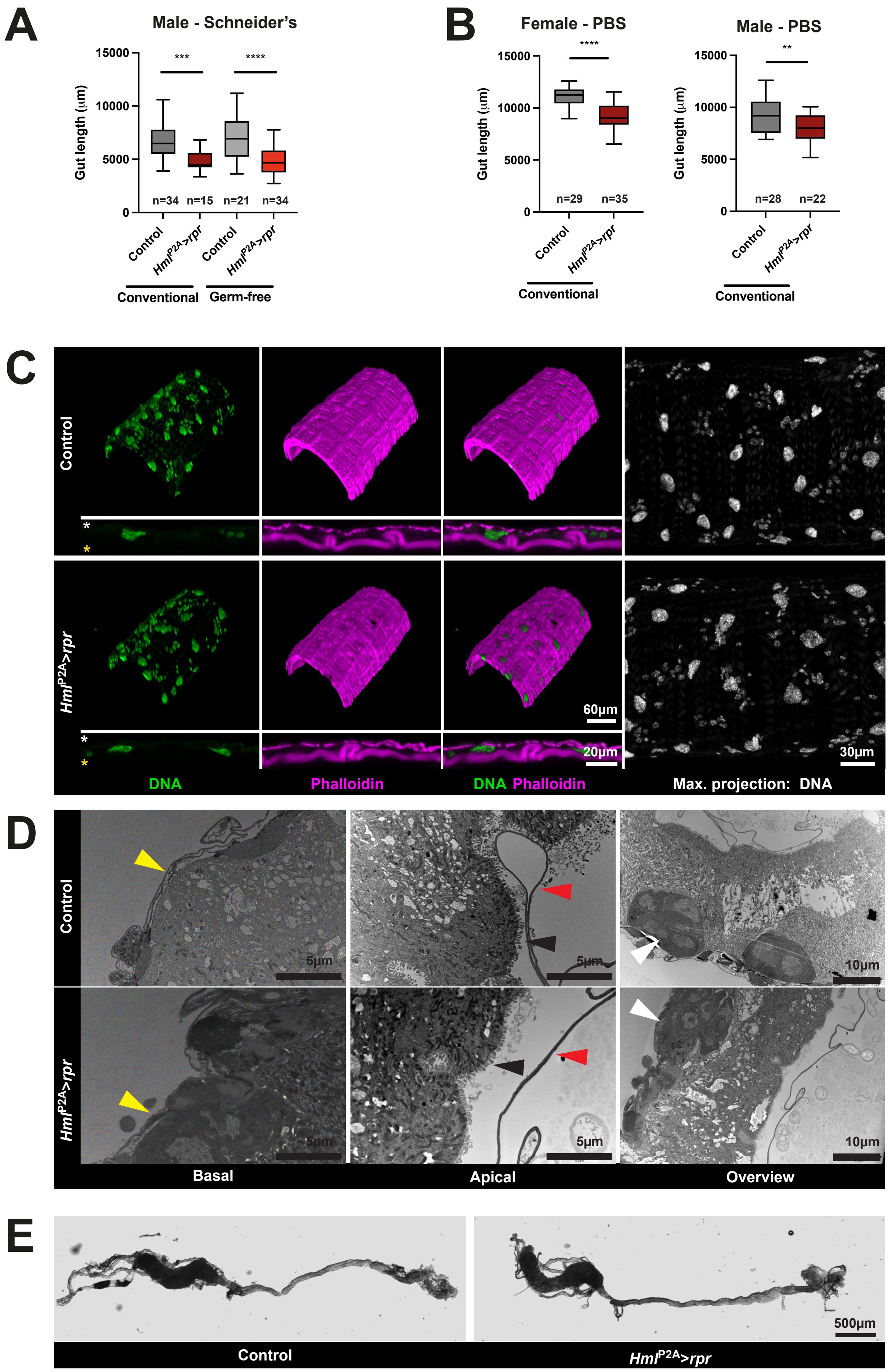

Development • Supplementary information

Fig. S6. (A & B) Midgut lengths from WS-larvae reared at controlled density were determined as outlined in Figure 4D. Dissection was carried out in Schneider's medium containing Ca^{2+} (A) or in calcium-free PBS (B), which reduced the contraction of guts after dissection. In both conditions, *Hml*^{P2A}>*rpr* (attP2) animals showed reduced midgut length compared to controls. This effect was observed despite the sexual dimorphism of midgut length in males and females and under conventional as well as germfree conditions. Number of guts are indicated. One-way ANOVA were performed. Box plot shows median values (middle bars) and first to third interquartile ranges (boxes); whiskers indicate minimum and maximum values.

(C). Fluorescent micrographs of midgut regions close to the midgut/hindgut border from *Hml*^{P2A}>*rpr* (attP2) and control WS-larvae reared at controlled density under conventional conditions and dissected in PBS. 3D reconstruction of DNA and Phalloidine staining consistently showed a slight deterioration of visceral musculature. In optical cross sections we observed no difference in staining the musculature on the basal side (white asterisk) and the brush border on the apical side of gut cells (yellow asterisk). Maximum projection of stacks visualized in 3D showed that enterocytes (large nuclei) and smaller cells that might include intestinal stem cells, enteroblasts and enterendocrine cells were present with no apparent differences.

(D) Electron micrographs of midgut regions close to the midgut/hindgut border from *Hml*^{P2A}>*rpr* (attP2) and control WS-larvae reared at controlled density under germ-free conditions. Basement membrane (yellow arrowheads), peritrophic membrane (red arrowheads) and microvilli (black arrowheads) are comparable between the genotypes.

(E) Brightfield images of pupal guts dissected from *Hml*^{P2A}>*rpr* (attP2) 24 h after pupariation and control pupae at 12h after pupariation.

Controls: yw>*rpr* (A-E). ** $p<0.002$; *** $p<0.0002$; **** $p<0.0001$

Table S1. RNA-seq data from HmlP2A>rpr (attP2) compared with attP2>rpr WS-larvae. Genes were classified as non-plasmatocyte (no or marginal expression in plasmatocytes), shared or plasmatocyte-enriched (>4 fold elevated in plasmatocytes).

Click here to download Table S1

Table S2. Genotypes derived from fly strains.

***reaper*-mediated ablation**

***Hml^{P2A}>rpr* (attP2):**

$w*/w^{1118}$; P{UAS-rpr.C}14/+; P{Hml-GAL4.P2A}attP2/+

***Hml^{P2A}>rpr* (attP40):**

$w*/w^{1118}$; P{UAS-rpr.C}14/ P{Hml-GAL4.P2A}attP40; +/+

***Hml^{P2A}>rpr* (attP40); *srp^{Hemo}>mCherry*:**

$w*/w^{1118}$; P{UAS-rpr.C}14/ P{Hml-GAL4.P2A}attP40;

P{10XQUAS-6XmCherry-HA}attP2/M{srpHemo-QF2}ZH-86Fb

***HmlΔ>rpr*:**

$w*/w^{1118}$; P{UAS-rpr.C}14/ P{Hml-GAL4.Delta}2; +/+

***Bax*-mediated ablation**

***Hml^{P2A}>Bax* (attP2):**

$w*/w^{1118}$; P{UAS-Bax.G} /+; P{Hml-GAL4.P2A}attP2/+

***Hml^{P2A}>Bal* (attP2), internal control genotype GFP+ and Cy-:**

$w*/w^{1118}$; CyO, *P{ActGFP}JMR1/+; P{Hml-GAL4.P2A}attP2/+*

***HmlΔ>Bax*:**

$w*/w^{1118}$; P{UAS-Bax.G}/P{Hml-GAL4.Delta}2; +/+

***HmlΔ>Bal*, internal control genotype GFP+ and Cy-:**

$w*/w^{1118}$; CyO, *P{ActGFP}JMR1/P{Hml-GAL4.Delta}2; +/+*

Expression analysis

***Hml^{P2A}>GFP* (attP2):**

$w*/w^{*}$; P{ UAS-2xEGFP}AH2/+; P{Hml-GAL4.P2A}attP2/+

***Hml^{P2A}>GFP* (attP40):**

$w*/w^{*}$; P{ UAS-2xEGFP}AH2/ P{Hml-GAL4.P2A}attP40; +/+

***Hml^{P2A}>GFP* (attP40); *srp^{Hemo}>mCherry*:**

$w*/w^{*}$; P{ UAS-2xEGFP}AH2/ P{Hml-GAL4.P2A}attP40;

P{10XQUAS-6XmCherry-HA}attP2/M{srpHemo-QF2}ZH-86Fb

***HmlΔ>GFP*:**

$w*/w^{*}$; P{ UAS-2xEGFP}AH2/P{Hml-GAL4.Delta}2; +/+

**Genetic rescue experiments**
Hml^{P2A}***>rpr*** **(attP2);** ***srp***^{Hemo}***-GAL80***:<br>*w*/w***; P{UAS-rpr.C}14/ +;*<br>*P{Hml-GAL4.P2A}attP2/M{ srpHemo-GAL80}ZH-86Fb*<br>***Hml***^{P2A}***>rpr*** **(attP2);** ***HmlΔ>GAL80***:<br>*w*/w***; P{UAS-rpr.C}14/P{Hml-QF2.Delta.L}2;*<br>*P{QUAS-GAL80.P}28/ P{Hml-GAL4.P2A}attP2*<br>***Hml***^{P2A}***>rpr*** **(attP40);** ***srp***^{Hemo}***>GAL80***:<br>*w*/w***; P{UAS-rpr.C}14/ P{Hml-GAL4.P2A}attP40;*<br>*P{QUAS-GAL80.P}28/M{srpHemo-QF2}ZH-86Fb*
**Control genotypes**
attP2>rpr **(attP2)**:<br>*w*/ y*^{1} *w*^{67c23}*; P{UAS-rpr.C}14/+; P{CaryP}attP2/+*<br>***yw>rpr*** **(attP2)**:<br>*w*/ y*^{1} *w***; P{UAS-rpr.C}14/+; +/+*

For male animals the second X chromosome was Y.

Development • Supplementary information
