# Supplementary material for: Hemocytes are essential for Drosophila melanogaster post-embryonic development, independent of control of the microbiota
Source: Development. 2022 Sep 29;149(18):dev200286. doi: 10.1242/dev.200286 (PMC9641648; doi:10.1242/dev.200286)
Supplement: Supplementary file 1 [file develop-149-200286_review_history.pdf]

Hemocytes are essential for *Drosophila melanogaster* post-embryonic development, independent of control of the microbiota

Holly N. Stephenson, Robert Streeck, Florian Grüblinger, Christian Goosmann and Alf Herzig

DOI: 10.1242/dev.200286

Editor: Paul Martin

Review timeline

Original submission:	21 October 2021
Editorial decision:	29 November 2021
First revision received:	7 July 2022
Accepted:	19 August 2022

Original submission

First decision letter

MS ID#: DEVELOP/2021/200286

MS TITLE: Haemocytes are critical for Drosophila melanogaster post-embryonic development, independent of control of the microbiota

AUTHORS: Holly N Stephenson, Robert Streeck, and Alf Herzig

I have now received all the referees' reports on the above manuscript, and have reached a decision. The referees' comments are appended below, or you can access them online: please go to BenchPress and click on the 'Manuscripts with Decisions' queue in the Author Area.

As you will see, the referees express considerable interest in your work, but have some significant criticisms and recommend a substantial revision of your manuscript before we can consider publication. If you are able to revise the manuscript along the lines suggested, which will involve further experiments, I will be happy receive a revised version of the manuscript. Your revised paper will be re-reviewed by one or more of the original referees, and acceptance of your manuscript will depend on your addressing satisfactorily the reviewers' major concerns. Please also note that Development will normally permit only one round of major revision.

We are aware that you may be experiencing disruption to the normal running of your lab that make experimental revisions challenging. If it would be helpful, we encourage you to contact us to discuss your revision in greater detail. Please send us a point-by-point response indicating where you are able to address concerns raised (either experimentally or by changes to the text) and where you will not be able to do so within the normal timeframe of a revision. We will then provide further guidance. Please also note that we are happy to extend revision timeframes as necessary.

Please attend to all of the reviewers' comments and ensure that you clearly highlight all changes made in the revised manuscript. Please avoid using 'Tracked changes' in Word files as these are lost in PDF conversion. I should be grateful if you would also provide a point-by-point response detailing how you have dealt with the points raised by the reviewers in the 'Response to Reviewers' box. If you do not agree with any of their criticisms or suggestions please explain clearly why this is so.

© 2022. Published by The Company of Biologists under the terms of the Creative Commons Attribution License (https://creativecommons.org/licenses/by/4.0/).

Reviewer 1

Advance summary and potential significance to field

In this study, Stephenson HN at al. provide data supporting a new insight into Drosophila developmental biology and hemocyte biology. Specifically, they argue that hemocytes are essential for metamorphosis because complete ablation of haemocytes leads to pupal lethality, an effect that is not due to their antimicrobial function.

Stephenson HN at al. have for the first time generated a hemocyte-specific GAL4 driver line based on the Hml gene which is strong enough to induce apoptosis of >99% of sessile and circulating haemocytes in Drosophila larvae. This is an extremely useful reagent and an advance for the field, given the many haemocyte-specific driver lines generated previously for different purposes including genetically-induced ablation, which have all had much lower efficiency. Using this construct, they were able to revisit the role of hemocytes in pupal development and have shown that contrary to previous findings, Drosophila don't eclose from pupae if there are no sessile or circulating hemocytes, even in a germ-free condition. Thus, they show for the first time that hemocytes have functions essential for development after the embryonic stage, which are independent of the presence of pathogens. This conclusion, if fully justified would be very interesting for the hemocyte and Drosophila development field and match the proven requirement for hemocytes during initial embryonic development.
Many further studies on interactions between hemocytes and tissues would be prompted. There are however some caveats to the conclusion based on the control utilized, rescuing eclosion with another hemocyte-specific driver (srp) (see below).

In addition, Stephenson HN at al. provide RNAseq analysis of sessile or circulating hemocyte-ablated larvae and using published FlyAtlas2 database have extracted tissue-specific changes in gene transcription after hemocyte ablation.
Based on this analysis they identified most changes as occurring in the midgut and postulate that the ablation of hemocytes likely causes abnormality in midgut development and hence pupal lethality. However, they did not provide any data on the structure or physiology of the midgut or other tissues during metamorphosis nor they provide any hint as to the direct cause of lethality. It remains unclear in which tissues a functional or developmental failure leads to lethality of pupae.

Overall, the paper is clear and well-written. However, at the moment it is closer to a resource paper than one providing substantive insight about development. As it is categorized as a Research Report, I will review it as such.

Comments for the author

Major points:

1) Rescue of the block in pupal development caused by Hml e9-P2A-GAL4 UAS-rpr by inhibiting GAL4 function in all hemocytes by srpHemo-80 or srpHemo-QF2 QUAS-GAL80 strongly supports the paper's main conclusion that the absence of hemocytes leading to the pupal defect. However some extraneous expression has been reported for the srpHemo-QF2 construct (Gyoergy et al 2018), in the fat body in the larva, and in the pupa this was not analyzed. Ablation of parts of the fat body could lead to changes in the midgut, due to effects on the amount of lipids in the hemolymph or other metabolic effects. To fully validate this control, the authors should assess, in the larva and (if the lethal phase falls within the pupal period) by pupal dissection, if srpHemo-QF2 and hml e9-P2A GAL4 (driving whatever QUAS or UAS they prefer) have any overlapping extraneous expression outside of hemocytes. If they do, the authors should try to find a GAL4 driver only expressing in this extraneous location and test if expressing reaper under that driver also causes pupal lethality. Without this, the main conclusion is not solidly substantiated
2) Pupa lethality data in antibiotic and germ-free conditions is central to the conclusions of the paper (Fig. 2CD). Some insight into reasons of the developmental arrest in those conditions should be included to meet the standard for Development papers to shed significant light on a developmental mechanism.
The authors should dissect non-eclosing pupae and characterize their stage or condition in some way to answer which tissue is most affected tissue by hemocyte ablation. Preferably compare

© 2022. Published by The Company of Biologists under the terms of the Creative Commons Attribution License (https://creativecommons.org/licenses/by/4.0/).

midgut structure of Hml-e9-P2A>rpr pupae to control. Does the midgut in hemoless pupae indeed contain more chitin as the authors speculate (line 230, line 98) or have other detectable defects?

3) The authors are vague as to when the lethality actually occurs. They should conduct a rigorous analysis of this. Do the authors see the same number of L2, L3, Wandering L3 larvae in Hml-e9-P2A>Bax vs Hml-e9-P2A>GFP and >Bax if equal numbers of L1 are seeded? Through which of the 20 morphologically recognizable pupal stages do they go? (See Bainbridge SP, Bownes M. Staging the metamorphosis of Drosophila melanogaster. J. Embryol. Exp. Morphol. 1981;66:57–80.) An understanding of when exactly the lethal pupal phase is would help clarify how relevant the RNAseq they conduct is in wandering third instar larvae. If the pupal lethality is just after pupariation, this RNAseq just prior could be appropriate. If it is much later, then pupal RNA seq would be more likely to reveal the reason for the lethality.

4) The authors appear to only analyze if sessile and circulating hemocytes are ablated upon Hml e9-P2A-GAL4 driver UAS-rpr *or UAS-Bax), as they bleed larvae after dislodging hemocytes with a paintbrush to conduct the analysis shown in 1D. To examine all hemocytes in the larvae, they should conduct FACS analysis after disrupting with a douncer to free all hemocytes, including those in tissues. This would be feasible by using Hml e9-P2A-GAL4 driver UAS-rpr UAS-GFP vs Hml e9-P2A-GAL4 driver UAS-lacZ UAS-GFP larvae or if they worry about dilution the effect through extra UASs, srpHemo-3xmCherry Hml e9-P2A-GAL4 driver UAS-rpr vs srpHemo-3xmCherry Hml e9-P2A-GAL4 driver UAS-GFP. With their current analysis, the possibility remains that a subpopulation of some adhesive hemocytes or ones that might lie within tissues, such as those seen in the fat body in the adult, are not ablated.

5) Mentioning the previous visualization of hemocytes in the fat body (Gyoergy, Roblek, et al. 2018) (Cox et al. Science 2021) would aid the reader in interpreting the data explained in lines 211-215. However, Fig.S3B (upper panel) shows that hemocytes themselves are predominantly in the carcass (lines 203-204). Please clarify how Fig.S3B fits to the conclusion about fat body transcripts (lines 211-216) and to the distribution of haemocytes in tissues.

6) Additionally from the graphs Fig. 2CD its visible that some Hml-e9-P2A>rpr and Hml-e9-P2A>Bax flies are able to eclose. Do these flies have some remaining hemocytes, or did they overcome the developmental/ physiological issues resulting from total hemocyte ablation? This question can be addressed by adding UAS-GFP to Hml-e9-P2A>rpr and imaging hemocytes in the eclosed flies.

If they have eclosed despite lacking hemocytes, characterizing their gut to determine if there is a correspondence between any structural defects they identified in point 1 and survival would clarify the relevance of changes they identify in point 1. If there are none, checking if these flies do not survive as well would strengthen the idea of there being a physiological deficit

7) The essential details for this paper are described in a yet unpublished manuscript, not listed in the References list. Therefore, either its citation (lines 117, 198) should contain a reference to the preprint on Biorxiv, and/or essential information on the driver construction and plasmatocyte-specific RNAseq should be included in the paper. Additionally, stating in the methods that the RNAseq libraries for this study were prepared and sequenced at Max Planck (line 368) is not sufficient; please specifically describe the method for RNA production, the sequencing approach, and the analysis.

8) Graphs in Fig. 2 and Fig S2C show “fraction of expected”, however what is taken as the “expected” value is not clear. Please add the definition to the Figure legends and potentially a longer explanation in the methods. Also address in the text why in the panels on the right side in Fig.2B,C,D the fraction of eclosion is much higher than “expected”.

9) Lines 140-142: substantiate this sentence about lamellocyte numbers by presenting corresponding data.

Questions regarding methods, this should be clarified in the text or corresponding methods sections:

10) Hemocyte extraction: Do hemocytes express GFP? What are the genotypes of the larvae used for extraction and counting?

11) Antibiotics, Germ-free conditions: are these methods identical or more/less efficient to the methods used in the previous studies, which show that hemocytes are mostly required to control microbiota in metamorphosis?

12) In Fig.3C: why are the upregulated gene groups visible for the Fat body, not present in the whole set “all”?

© 2022. Published by The Company of Biologists under the terms of the Creative Commons Attribution License (https://creativecommons.org/licenses/by/4.0/).

Minor points/ textual comments:
13) Line 43: Add recent study on plasmatocyte subpopulations to references:
Coates JA, Brooks E, Brittle AL, Armitage EL, Zeidler MP, Evans IR.
Identification of functionally distinct macrophage subpopulations in Drosophila.
Elife. 2021 Apr 22;10:e58686. doi: 10.7554/eLife.58686. PMID: 33885361; PMCID: PMC8062135.
14) Fig.2A: provide statistical analysis.
15) Line 161: the word “lower” is ambiguous – reduction is lower or eclosion rate is lower?
16) Line 193, add wandering third instar larvae.
17) Fig.2B right: provide statistical analysis for comparison of gray and blue datasets.
18) Fig. S2A,B: I believe you meant to write Hml-e9-P2A>GFP instead of attp2 >GFP
19) Fig. S2A,B: add statistical analysis
20) Fig.3D: explain more clearly in the Figure and in the Figure legend that what is shown on the graph is the tissue enrichment measured in intact larvae for those genes which are depleted after hemocyte ablation.
21) Fig. S3C: how does this graph substantiate the sentence in line 205.
Explain what PCA analysis shows and the 4 dots of each color represent.

Reviewer 2

Advance summary and potential significance to field

This study reports the development of a new GAL4 line for conditional expression of transgenes in Drosophila larval hemocytes and use of this line to demonstrate that apoptotic ablation of hemocytes results in pupal death, thus suggesting that hemocytes are essential for pupal metamorphosis. Bioinformatic analysis of gene expression in hemocyte-depleted larvae suggests that the gut is perturbed following hemocyte depletion.

The conclusion that hemocytes are required for pupal morphogenesis is novel and interesting. If correct, this finding would open an interesting new area that could reveal important new functions for hemocytes and possibly immune cells more generally. In addition, the new GAL4 line described in the report appears to be an excellent new tool that would likely be useful to other researchers. This work is therefore potentially of interest to the community. In its current form, this study is light on experimental data and in particular a little more data is required to conclusively prove that hemocytes are required for eclosion. The gene expression analysis is interesting, but it is impossible to know from the data presented whether it has any relevance to the central finding of the paper, i.e the pupal lethality of hemocyte ablation.

Comments for the author

Major points:
1. This study only really has one clear conclusion: that hemocytes are required for eclosion. The evidence supporting this conclusion therefore needs to be very convincing. The data as presented to support this point is good, but incomplete. Firstly, the experimental characterisation of the new GAL4 line is quite limited. Given that the main conclusion of the manuscript rests on this line being hemocyte-specific and that the development of this GAL4 line is a key aspect of the study, more detailed characterisation of its expression pattern is required. What is the developmental expression profile of this GAL4 line? At present expression in L3 larvae and adults is shown. Is expression detected in other developmental stages? Does the expression pattern perfectly reproduce that of other hemocyte drivers and markers throughout development? Secondly, it would be reassuring to know that expression of GAL80 with a strong non-hemocyte driver doesn’t rescue eclosion when rpr is expressed in hemocytes.
2. The manuscript has no experiments that address the mechanism by which hemocyte depletion results in failure of eclosion. While a detailed mechanistic analysis of this is probably beyond the scope of the study, given that the failure of eclosion is the central finding of the study, I think some investigation of this is required. For example, it would be relatively straightforward to determine how far pupation proceeds and whether there are any obvious morphological defects in dead pupae.

© 2022. Published by The Company of Biologists under the terms of the Creative Commons Attribution License (https://creativecommons.org/licenses/by/4.0/).

3. On a similar note, based on bioinformatic analysis the authors suggest that ECM in the larval gut or gut integrity may be affected by hemocyte depletion, but there are no experiments to test this hypothesis or validate the bioinformatic analysis. It would be relatively straightforward to examine the morphology and/or ECM composition of the L3 gut to obtain some evidence to support the hypothesis that the gut is in some way perturbed by hemocyte depletion.
4. In this study hemocytes are depleted by inducing apoptosis. Is it possible that the changes in gene expression (and potentially pupal death) are a result of a systemic response to the presence of a large population of apoptotic cells, rather than the absence of hemocytes? Potential drawbacks of the approach used and caveats in interpreting the data should be discussed.

Minor points:
1. AMP should be defined on first use.
2. The authors may wish to consider using the spelling ‘hemocyte’, which is used far more widely in the Drosophila melanogaster community than ‘haemocyte’. I’m not sure if CoB have a policy on which should be used, but I think it would be helpful for the community to move towards adopting a consistent spelling.

First revision

Author response to reviewers' comments

Reviewer 1 Comments for the author
Major points:

1) Rescue of the block in pupal development caused by Hml e9-P2A-GAL4 UAS-rpr by inhibiting GAL4 function in all hemocytes by srpHemo-80 or srpHemo-QF2 QUAS-GAL80 strongly supports the paper’s main conclusion that the absence of hemocytes leading to the pupal defect. However some extraneous expression has been reported for the srpHemo-QF2 construct (Gyoergy et al 2018), in the fat body in the larva, and in the pupa this was not analyzed. Ablation of parts of the fat body could lead to changes in the midgut, due to effects on the amount of lipids in the hemolymph or other metabolic effects. To fully validate this control, the authors should assess, in the larva and (if the lethal phase falls within the pupal period) by pupal dissection, if srpHemo-QF2 and hml e9-P2A GAL4 (driving whatever QUAS or UAS they prefer) have any overlapping extraneous expression outside of hemocytes. If they do, the authors should try to find a GAL4 driver only expressing in this extraneous location and test if expressing reaper under that driver also causes pupal lethality. Without this, the main conclusion is not solidly substantiated

This is a very important point and in fact central to our study. We have carried out a detailed analysis of srpHemo expression throughout development (Fig. 1; Fig. S2). We found prominent extraneous expression of the srpHemo-QF driver in pericardial nephrocytes and garland cells, consistent with published results (Das et al. 2008), but none of these domains overlapped with HmlP2A expression. We would also like to point out that inhibiting GAL4 function by Hmldelta rescued lethality as well. This indicates that HmlP2A did not gain novel tissue expression compared to Hmldelta which is relevant for lethality. Hmldelta has been intensely used by the community and no expression outside the hematopoietic system has been reported.

2) Pupa lethality data in antibiotic and germ-free conditions is central to the conclusions of the paper (Fig. 2CD). Some insight into reasons of the developmental arrest in those conditions should be included to meet the standard for Development papers to shed significant light on a developmental mechanism.
The authors should dissect non-eclosing pupae and characterize their stage or condition in some way to answer which tissue is most affected tissue by hemocyte ablation. Preferably compare midgut structure of Hml-e9-P2A>rpr pupae to control. Does the midgut in hemoless pupae indeed contain more chitin as the authors speculate (line 230, line 98) or have other detectable defects?

We agree with the reviewer that is was important to better characterise the developmental arrest. We found that the arrest occurs around pupation in early pupal development. At this stage we

© 2022. Published by The Company of Biologists under the terms of the Creative Commons Attribution License (https://creativecommons.org/licenses/by/4.0/).

found multiple defective processes (leg/wing eversion, formation of white malpigian tubules, anterior moving ‘bubble’ and anterior detachment of the pupae). Unfortunately, therefore, we were unable to pinpoint a single tissue that was most affected by hemocyte ablation. On the contrary, we observed pupariation phenotypes and in ‘escapers’ wing inflation phenotypes (Fig. S4). We therefore believe that the phenotype caused by hemocyte ablation is highly pleiotropic and a thorough analysis would go beyond the scope of this paper. We removed our speculation about chitin.

3) The authors are vague as to when the lethality actually occurs. They should conduct a rigorous analysis of this. Do the authors see the same number of L2, L3, Wandering L3 larvae in Hml-e9-P2A>Bax vs Hml-e9-P2A>GFP and >Bax if equal numbers of L1 are seeded? Through which of the 20 morphologically recognizable pupal stages do they go? (See Bainbridge SP, Bownes M. Staging the metamorphosis of Drosophila melanogaster. J. Embryol. Exp. Morphol. 1981;66:57–80.) An understanding of when exactly the lethal pupal phase is would help clarify how relevant the RNAseq they conduct is in wandering third instar larvae. If the pupal lethality is just after pupariation, this RNAseq just prior could be appropriate. If it is much later, then pupal RNA seq would be more likely to reveal the reason for the lethality.

We agree the original data did not time lethality sufficiently. In our previous experiments (updated Fig. 3A, B and Fig. S3A) and also in newly included experiments (Fig. 3C, D and Fig. S3C) we found no effect on larval survival. In the new experiments we additionally used synchronized 1st instar larva (that hatched within a 5 h time window) for seeding and found only a slight extension of larval development under conventional conditions and no delay under germ-free conditions. With the driver line used for the RNAseq experiment we found highly penetrant (95%) pupal lethality <24 h after pupariation, which occurred based on our dissection around pupation in pupal stage P5. With our second driver line the phenotype was less expressive and pupal development went beyond that stage in some animals, which we quantified (Figs. 3C,D and S3C).

4) The authors appear to only analyze if sessile and circulating hemocytes are ablated upon Hml e9-P2A-GAL4 driver UAS-rpr *or UAS-Bax), as they bleed larvae after dislodging hemocytes with a paintbrush to conduct the analysis shown in 1D. To examine all hemocytes in the larvae, they should conduct FACS analysis after disrupting with a douncer to free all hemocytes, including those in tissues. This would be feasible by using Hml e9-P2A-GAL4 driver UAS-rpr UAS-GFP vs Hml e9-P2A-GAL4 driver UAS-lacZ UAS-GFP larvae or if they worry about dilution the effect through extra UASs, srpHemo-3xmCherry Hml e9-P2A-GAL4 driver UAS-rpr vs srpHemo-3xmCherry Hml e9-P2A-GAL4 driver UAS-GFP. With their current analysis, the possibility remains that a subpopulation of some adhesive hemocytes or ones that might lie within tissues, such as those seen in the fat body in the adult, are not ablated.

Thank you for suggesting this experiment. We now included a similar experiment comparing HmlP2A>rpr; srpHemo-QF>mCherry animals to HmlP2A>GFP; srpHemo-QF>mCherry (Fig. 2E). We found that srpHemo-QF>mCherry had greatly superior fluorophore expression compared to srpHemo-3xmCherry transgenes. We did not find traces of tissue resident hemocytes.

5) Mentioning the previous visualization of hemocytes in the fat body (Gyoergy, Roblek, et al. 2018) (Cox et al. Science 2021) would aid the reader in interpreting the data explained in lines 211-215. However, Fig.S3B (upper panel) shows that hemocytes themselves are predominantly in the carcass (lines 203-204). Please clarify how Fig.S3B fits to the conclusion about fat body transcripts (lines 211-216) and to the distribution of haemocytes in tissues.

Due to this comment and the increased amount of data in the paper we have significantly altered this section and clarified our conclusions. Based on the comparison between plasmatocyte and whole larval transcriptomes we identified plasmatocyte-enriched transcripts (Fig. 4A). These transcripts are found to be most highly enriched in the carcass (Fig. S5B). However, they are also found in other tissues but with lower enrichment. This probably reflects the situation that the majority of hemocytes are still associated with the carcass (cuticle, lymph gland).

6) Additionally from the graphs Fig. 2CD its visible that some Hml-e9-P2A>rpr and Hml-e9-P2A>Bax flies are able to eclose. Do these flies have some remaining hemocytes, or did they overcome the

© 2022. Published by The Company of Biologists under the terms of the Creative Commons Attribution License (https://creativecommons.org/licenses/by/4.0/).

developmental/ physiological issues resulting from total hemocyte ablation? This question can be addressed by adding UAS-GFP to Hml-e9-P2A>rpr and imaging hemocytes in the eclosed flies. If they have eclosed despite lacking hemocytes, characterizing their gut to determine if there is a correspondence between any structural defects they identified in point 1 and survival would clarify the relevance of changes they identify in point 1. If there are none, checking if these flies do not survive as well would strengthen the idea of there being a physiological deficit

Thank you for this observation. We assessed the presence of plasmatocytes in HmlP2A>rpr; srpHemo-QF>mCherry adult escapers and found them to be present (Fig. S4D).

7) The essential details for this paper are described in a yet unpublished manuscript, not listed in the References list. Therefore, either its citation (lines 117, 198) should contain a reference to the preprint on Biorxiv, and/or essential information on the driver construction and plasmatocyte-specific RNAseq should be included in the paper. Additionally, stating in the methods that the RNAseq libraries for this study were prepared and sequenced at Max Planck (line 368) is not sufficient; please specifically describe the method for RNA production, the sequencing approach, and the analysis.

We have included the RNAseq data for plasmatocytes in this manuscript now. This includes materials and methods as well as raw data in the data availability section. We also amended the methods section for sequencing details. While the RNAseq data is without doubt critical for the conclusions of the paper, the ChIPseq data that we showed in Fig. 1 is not. Therefore, we removed it from the paper.

8) Graphs in Fig. 2 and Fig S2C show “fraction of expected”, however what is taken as the “expected” value is not clear. Please add the definition to the Figure legends and potentially a longer explanation in the methods. Also address in the text why in the panels on the right side in Fig.2B,C,D the fraction of eclosion is much higher than “expected”.

We agree that this needed clarification and we amended the figure legends and methods section accordingly. For the experiments in shown in updated Fig. S4A & B we used the homozygous lethal UAS-Bax/CyO, Act-GFP line crossed to a homozygous HmlP2A-GAL4 line. From this cross we expected 50% GFP negative pupae or 50% Cy+ adults (HmlP2A>Bax) if there was no effect in Bax expressing animals. The fraction of expected therefore was scored relative to balancer carrying animals. Since balancer chromosomes can to some degree reduce fitness, the fraction of expected can be higher than 1 in some cases.

9) Lines 140-142: substantiate this sentence about lamellocyte numbers by presenting corresponding data.

Thank you for this comment. We have included a new section on hemocyte quantification now (Fig. 2C). Lamellocytes were identified by their unique morphology and positive staining for the pan-hemocyte marker Hemese.

Questions regarding methods, this should be clarified in the text or corresponding methods sections:

10) Hemocyte extraction: Do hemocytes express GFP? What are the genotypes of the larvae used for extraction and counting?

We agree that this needed clarification. The original data were based on whole blood cell counts (which are predominantly plasmatocytes) in the absence of UAS-GFP or another marker to ensure that we did not dilute the effect of the GAL4 driver. We also wanted to provide an unbiased analysis of hemocytes by morphology rather than relying on a driver than may not express in all hemocyte subsets. We now have included a new section on hemocyte quantification using antibody stainings (Fig. 2C) to complement the original data. We also amended the materials and methods section for a summary of the genotypes used in the paper.

© 2022. Published by The Company of Biologists under the terms of the Creative Commons Attribution License (https://creativecommons.org/licenses/by/4.0/).

11) Antibiotics, Germ-free conditions: are these methods identical or more/less efficient to the methods used in the previous studies, which show that hemocytes are mostly required to control microbiota in metamorphosis?

We used antibiotic conditions that were identical to previous studies (Charroux et al. 2009), and generated germ-free flies by bleaching embryos which was also previously reported (Defaye et al. 2009). We amended the text to state this and have included a more detailed description of how we confirmed germ-free status in the materials & methods section (lines 337-342).

12) In Fig.3C: why are the upregulated gene groups visible for the Fat body, not present in the whole set “all”?

We agree that this was confusing and we greatly simplified this section. In the original version of the paper this was because the GO terms that reached significance in the “fat-body” subset did not reach significance in the whole set “all” which included more genes.

Minor points/ textual comments:

13) Line 43: Add recent study on plasmatocyte subpopulations to references:
Coates JA, Brooks E, Brittle AL, Armitage EL, Zeidler MP, Evans IR.
Identification of functionally distinct macrophage subpopulations in Drosophila.
Elife. 2021 Apr 22;10:e58686. doi: 10.7554/eLife.58686. PMID: 33885361; PMCID: PMC8062135.

14) Fig.2A: provide statistical analysis.

15) Line 161: the word “lower” is ambiguous – reduction is lower or eclosion rate is lower?

16) Line 193, add wandering third instar larvae.

17) Fig.2B right: provide statistical analysis for comparison of gray and blue datasets.

18) Fig. S2A,B: I believe you meant to write Hml-e9-P2A > GFP instead of attp2 > GFP

19)Fig. S2A,B: add statistical analysis

20) Fig.3D: explain more clearly in the Figure and in the Figure legend that what is shown on the graph is the tissue enrichment measured in intact larvae for those genes which are depleted after hemocyte ablation.

21) Fig. S3C: how does this graph substantiate the sentence in line 205.
Explain what PCA analysis shows and the 4 dots of each color represent.

We have made the above minor corrections to the manuscript.

Reviewer 2 Comments for the author
Major points:

1. This study only really has one clear conclusion: that hemocytes are required for eclosion. The evidence supporting this conclusion therefore needs to be very convincing. The data as presented to support this point is good, but incomplete. Firstly, the experimental characterisation of the new GAL4 line is quite limited. Given that the main conclusion of the manuscript rests on this line being hemocyte-specific and that the development of this GAL4 line is a key aspect of the study, more detailed characterisation of its expression pattern is required. What is the developmental expression profile of this GAL4 line? At present expression in L3 larvae and adults is shown. Is expression detected in other developmental stages? Does the expression pattern perfectly reproduce that of other hemocyte drivers and markers throughout development? Secondly, it would be reassuring to know that expression of GAL80 with a strong non-hemocyte driver doesn’t rescue eclosion when rpr is expressed in hemocytes.

© 2022. Published by The Company of Biologists under the terms of the Creative Commons Attribution License (https://creativecommons.org/licenses/by/4.0/).

We agree with the reviewer in that our initial characterizations of the HmlP2A lines were incomplete. We have now performed a comprehensive expression analysis of HmlP2A >GFP in embryos, 1st, 2nd & 3rd instar larvae and adults by fluorescence microscopy and compared it to srpHemo>mCherry expression (Fig. 1, Fig. S2). We did not find HmlP2A>GFP expression outside the hematopoietic system.

2. The manuscript has no experiments that address the mechanism by which hemocyte depletion results in failure of eclosion. While a detailed mechanistic analysis of this is probably beyond the scope of the study, given that the failure of eclosion is the central finding of the study, I think some investigation of this is required. For example, it would be relatively straightforward to determine how far pupation proceeds and whether there are any obvious morphological defects in dead pupae.

Again, we agree that this point is very important, as reviewer 1 points 2 & 3 raised a similar question. We quantified pupal lethality for both driver lines (in attP2 or attP40) by establishing categories; lethality before stage P8, between stage P8-P14 or in stage P15 (Figs. 3C,D and S3C). For the driver line in attP2 we found highly penetrant (95%) pupal lethality within the first 24h after pupariation, which we placed by morphological criteria around pupation in stage P5.

3. On a similar note, based on bioinformatic analysis the authors suggest that ECM in the larval gut or gut integrity may be affected by hemocyte depletion, but there are no experiments to test this hypothesis or validate the bioinformatic analysis. It would be relatively straightforward to examine the morphology and/or ECM composition of the L3 gut to obtain some evidence to support the hypothesis that the gut is in some way perturbed by hemocyte depletion.

Thank you for this suggestion. We analysed guts from L3 larvae reared under conventional or germ-free conditions. We found that hemocyte ablation leads to a significantly reduced gut size in both conditions (Fig. 4D and Fig. S6A, B). Unfortunately, we did not see a clear difference in midgut structure by immunofluorescence or electron microscopy (Fig. S6C, D)

4. In this study hemocytes are depleted by inducing apoptosis. Is it possible that the changes in gene expression (and potentially pupal death) are a result of a systemic response to the presence of a large population of apoptotic cells, rather than the absence of hemocytes? Potential drawbacks of the approach used and caveats in interpreting the data should be discussed.

We agree that this is a possibility and an inherent problem with ablation experiments. We amended the text to include the statement "A drawback of apoptosis-driven depletion of hemocytes is that the increase in apoptotic bodies may in itself induce a physiological response in the fly, including upregulation of immune-related genes (Arefin et al., 2015). In future studies it would be interesting to assess whether the genetic manipulation of specific hemocyte functions recapitulates pupal lethality, as seen when hemocyte migration is impaired in embryos (Matsubayashi et al., 2017)." (Lines 223 – 227)

Minor points:
1. AMP should be defined on first use.

This has been amended

2. The authors may wish to consider using the spelling 'hemocyte', which is used far more widely in the Drosophila melanogaster community than 'haemocyte'. I'm not sure if CoB have a policy on which should be used, but I think it would be helpful for the community to move towards adopting a consistent spelling.

We have changed the spelling to 'hemocyte'.

© 2022. Published by The Company of Biologists under the terms of the Creative Commons Attribution License (https://creativecommons.org/licenses/by/4.0/).

Second decision letter

MS ID#: DEVELOP/2021/200286

MS TITLE: Hemocytes are critical for Drosophila melanogaster post-embryonic development, independent of control of the microbiota

AUTHORS: Holly N Stephenson, Robert Streeck, Alf Herzig, Florian Grueblinger, and Christian Goosmann
ARTICLE TYPE: Research Report

I am happy to tell you that your manuscript has been accepted for publication in Development, pending our standard ethics checks. , and we would like you to address the minor comments that one of them raises and make sure that you have the paper proof checked for grammar/spellings.

Reviewer 1

Advance summary and potential significance to field

The manuscript is greatly improved and now provides a convincing case that Drosophila hemocytes are required for multiple developmental processes during larval and pupal development independent of bacterial infection.
It also describes a new hemocyte driver which is capable of expressing strongly in almost all hemocytes, enabling nearly complete ablation of this cell type.

Comments for the author

There are a few small remaining points that would improve the manuscript further.
Experimental:
1) Regarding Previous point 2:
The authors state in lines 171-5 ("We confirmed by dissection that legs/wings were partially everted, but malpigian tubules had not yet formed compact white structures as seen in controls 24 h post-pupariation." However they provide no corresponding images. Could the authors not show images of the phenotype which could prove informative to those interested in gut and pupal development?
Textual:
2) Regarding Previous point 8:
Adding the very clear explanation the authors provided in their response to the materials and methods would be beneficial for the reader:
"For the experiments in shown in updated Fig. S4A & B we used the homozygous lethal UAS-Bax/CyO, Act-GFP line crossed to a homozygous HmlP2A-GAL4 line. From this cross we expected 50% GFP negative pupae or 50% Cy+ adults (HmlP2A>Bax) if there was no effect in Bax expressing animals. The fraction of expected therefore was scored relative to balancer carrying animals. Since balancer chromosomes can to some degree reduce fitness, the fraction of expected can be higher than 1 in some cases. "
to the materials and methods.
3) Regarding Previous point 9 (re lamellocytes):
I may have missed it, but I did not find a description in the methods of what the authors used as criteria to determine when counting cells which of them were lamellocytes (size above a certain threshold etc.). Please add this if it is not present.
4) A suggestion: The color scheme in Figure 4B distinguishing differential from non differential is subtle. Is it possible to have the colors sets be more strikingly distinct?
5) Having a native English speaker proofread the added text would be helpful. There were minor grammatical errors. Phalloidin was also misspelled as phalloidine.
Note: It would have been helpful if the authors had always indicated in their reviewer response the precise line numbers of their included changes and provided a manuscript with textual changes highlighted in another color.

© 2022. Published by The Company of Biologists under the terms of the Creative Commons Attribution License (https://creativecommons.org/licenses/by/4.0/).

Reviewer 2

Advance summary and potential significance to field

See comments for previous version.

Comments for the author

The new data is of a high standard and addresses my previous concerns very well. I am therefore happy to recommend publication of this manuscript, although I have one minor suggestion: The data regarding gut length is interesting and helps integrate the gene expression data into the rest of the manuscript, however this result is not discussed at all. Could the authors perhaps very briefly comment on what the reduced gut length in hemoless larvae might suggest about hemocyte function in gut morphogenesis/maintenance/function?

Second revision

Author response to reviewers' comments

Response to the reviewer comments:

Reviewer 1

Experimental:

1) Regarding Previous point 2:
The authors state in lines 171-5 ("We confirmed by dissection that legs/wings were partially everted, but malpigian tubules had not yet formed compact white structures as seen in controls 24 h post-pupariation." However they provide no corresponding images. Could the authors not show images of the phenotype which could prove informative to those interested in gut and pupal development?

Our main conclusion about the stage of pupal lethality is based on data that we quantified and provided images for in Figure S4E (text: lines 170-173). The observations Reviewer 1 is referring to are supplementary to this conclusion. Unfortunately, we do not have images of these secondary observations (our primary focus was to document the gut phenotype in the respective pupae, Figure S6E). We believe that the data presented is sufficient to support our conclusions and that the extra value added by taking these pictures would not warrant the delay of ~2months for experimentation, especially since the images would not reveal more detail than already stated in the text. We do not read the question posed by the reviewer as a strict requirement to add these images, but please let us know if you do believe them to be essential or if you would like us to remove these secondary observations from the text.

Textual:
2) Regarding Previous point 8:
Adding the very clear explanation the authors provided in their response to the materials and methods would be beneficial for the reader:
"For the experiments in shown in updated Fig. S4A & B we used the homozygous lethal UAS-Bax/CyO, Act-GFP line crossed to a homozygous HmlP2A-GAL4 line. From this cross we expected 50% GFP negative pupae or 50% Cy+ adults (HmlP2A>Bax) if there was no effect in Bax expressing animals. The fraction of expected therefore was scored relative to balancer carrying animals. Since balancer chromosomes can to some degree reduce fitness, the fraction of expected can be higher than 1 in some cases. "
to the materials and methods.

We have added additional text, lines 329-333, to the M&M section so it now reads "For crosses involving *P{UAS-Bax.G}*, pupal survival was scored as number of adults relative to internal

© 2022. Published by The Company of Biologists under the terms of the Creative Commons Attribution License (https://creativecommons.org/licenses/by/4.0/).

control genotype (*Hml*^{P2A}>*Bax per Hml*^{P2A}>*Bal*). From this cross we expected 1:1 *Hml*^{P2A}>*Bax : Hml*^{P2A}>*Bal* adults if there was no effect in *Bax* expressing animals. The fraction of expected therefore was scored relative to balancer carrying animals. Since balancer chromosomes can to some degree reduce fitness, the fraction of expected can be higher than 1 in some cases."

3) Regarding Previous point 9 (re lamellocytes):
I may have missed it, but I did not find a description in the methods of what the authors used as criteria to determine when counting cells which of them were lamellocytes (size above a certain threshold etc.). Please add this if it is not present.

We have added to following text to the M&M section, lines 376-379, "Lamellocytes were selected manually from scans of entire wells stained with anti-Hemese antibody and checked for a minimum single cell size of 300 µm^2, which was ~2.5 times larger than the average size of Hemese positive cells (~120 µm^2)."

4) A suggestion: The color scheme in Figure 4B distinguishing differential from non differential is subtle. Is it possible to have the colors sets be more strikingly distinct?

We have modified the shading of the differential/non differential to make the difference more obvious. We would like to keep the original colour scheme as the genes, whether differentially expressed or not, are in the same group of genes (plasmatocyte enriched, shared or non-plasmatocyte).

5) Having a native English speaker proofread the added text would be helpful. There were minor grammatical errors. Phalloidin was also misspelled as phalloidine.

We have had a native English speaker check the manuscript and have altered any grammatical errors.

Reviewer 2:

The new data is of a high standard and addresses my previous concerns very well. I am therefore happy to recommend publication of this manuscript, although I have one minor suggestion: The data regarding gut length is interesting and helps integrate the gene expression data into the rest of the manuscript, however this result is not discussed at all. Could the authors perhaps very briefly comment on what the reduced gut length in hemoless larvae might suggest about hemocyte function in gut morphogenesis/maintenance/function?

Due to the word limit we minimised our discussion of hemocyte functions during morphogenesis in the manuscript. We have now expanded this slightly to read "One of these studies showed that hemocyte-derived signalling promotes basal and damage/infection induced proliferation of intestinal stem cells in adults but failed to detect a larval gut phenotype after ablation of hemocytes with *Hml*$^{\Delta}$ (Ayyaz et al., 2015). The striking reduction in larval midgut length that we observed could indicate a similar developmental function of hemocytes, which was previously obscured by incomplete hemocyte ablation. However, we cannot exclude that lethality resulted from pleiotropic defects that we observed later in development." Lines 224-230.

© 2022. Published by The Company of Biologists under the terms of the Creative Commons Attribution License (https://creativecommons.org/licenses/by/4.0/).
